# Wearable Devices to Improve Physical Activity and Reduce Sedentary Behaviour: An Umbrella Review

**DOI:** 10.1186/s40798-024-00678-9

**Published:** 2024-01-14

**Authors:** Jessica Longhini, Chiara Marzaro, Silvia Bargeri, Alvisa Palese, Andrea Dell’Isola, Andrea Turolla, Paolo Pillastrini, Simone Battista, Greta Castellini, Chad Cook, Silvia Gianola, Giacomo Rossettini

**Affiliations:** 1 Department of Diagnostics and Public Health, University of Verona, Verona, Italy; 2 Vicenza, Italy; 3 Unit of Clinical Epidemiology, IRCCS Istituto Ortopedico Galeazzi, Milan, Italy; 4 Department of Clinical Sciences Lund, Clinical Epidemiology Unit, Orthopedics, Lund University, Lund, Sweden; 5 Department of Biomedical and Neuromotor Sciences (DIBINEM), Alma Mater Studiorum Università di Bologna, Bologna, Italy; 6 Unit of Occupational Medicine, IRCCS Azienda Ospedaliero-Universitaria di Bologna, Bologna, Italy; 7 Department of Neurosciences, Rehabilitation, Ophthalmology, Genetics, Maternal and Child Health, University of Genoa, Campus of Savona, Savona, Italy; 8 Department of Orthopaedics, Division of Physical Therapy, Duke University, Durham, NC USA; 9 School of Physiotherapy, University of Verona, Verona, Italy; 10 Department of Human Neurosciences, University of Roma “Sapienza Roma”, Rome, Italy; 11 Department of Physiotherapy, Faculty of Sport Sciences, Universidad Europea de Madrid, Calle Tajo s/n, Villaviciosa de Odón 28670, Spain

**Keywords:** Wearable devices, Activity monitors, Physical activity, Sedentary behaviour, Osteoarthritis, Low back pain, Musculoskeletal, Obesity, Hypertension, Frailty

## Abstract

**Background:**

Several systematic reviews (SRs), with and without meta-analyses, have investigated the use of wearable devices to improve physical activity, and there is a need for frequent and updated syntheses on the topic.

**Objective:**

We aimed to evaluate whether using wearable devices increased physical activity and reduced sedentary behaviour in adults.

**Methods:**

We conducted an umbrella review searching PubMed, Cumulative Index to Nursing and Allied Health Literature, the Cochrane Library, MedRxiv, Rxiv and bioRxiv databases up to February 5th, 2023. We included all SRs that evaluated the efficacy of interventions when wearable devices were used to measure physical activity in adults aged over 18 years. The primary outcomes were physical activity and sedentary behaviour measured as the number of steps per day, minutes of moderate to vigorous physical activity (MVPA) per week, and minutes of sedentary behaviour (SB) per day. We assessed the methodological quality of each SR using the Assessment of Multiple Systematic Reviews, version 2 (AMSTAR 2) and the certainty of evidence of each outcome measure using the GRADE (Grading of Recommendations, Assessment, Development, and Evaluations). We interpreted the results using a decision-making framework examining the clinical relevance and the concordances or discordances of the SR effect size.

**Results:**

Fifty-one SRs were included, of which 38 included meta-analyses (302 unique primary studies). Of the included SRs, 72.5% were rated as ‘critically low methodological quality’. Overall, with a slight overlap of primary studies (corrected cover area: 3.87% for steps per day, 3.12% for MVPA, 4.06% for SB) and low-to-moderate certainty of the evidence, the use of WDs may increase PA by a median of 1,312.23 (IQR 627–1854) steps per day and 57.8 (IQR 37.7 to 107.3) minutes per week of MVPA. Uncertainty is present for PA in pathologies and older adults subgroups and for SB in mixed and older adults subgroups (large confidence intervals).

**Conclusions:**

Our findings suggest that the use of WDs may increase physical activity in middle-aged adults. Further studies are needed to investigate the effects of using WDs on specific subgroups (such as pathologies and older adults) in different follow-up lengths, and the role of other intervention components.

**Supplementary Information:**

The online version contains supplementary material available at 10.1186/s40798-024-00678-9.

## Key Points

- There is a moderate certainty of evidence in our umbrella review, which included 51 systematic reviews (of which 38 included meta-analyses with 302 unique primary studies);
- Available evidence suggests that using wearable devices may effectively increase physical activity across different population in number of steps per day and in minutes spent from moderate to vigorous physical activity per week;
- Results on efficacy of wearable devices on minutes of sedentary behaviour per day are inconsistent.

**Supplementary Information:**

The online version contains supplementary material available at 10.1186/s40798-024-00678-9.

## Background

Physical activity (PA) is described as 'any body movement produced by skeletal muscles that requires energy expenditure', including activities performed at work, play, housework, travel, and recreation [1]. It provides health benefits, including prevention and treatment of many conditions such as osteoarthritis, low back pain, hypertension, stroke, obesity, diabetes, and mental health disorders (i.e., distress, anxiety and symptoms of depression both in healthy and ill adults) [1, 2]. There is also evidence that the risk of frailty might be reduced by modifying levels of PA [3].

Almost 30% of adults do not follow PA recommendations [4]. Failure to meet PA recommendations is likely influenced by a modern society that promotes long-term sitting during free time, work, and commuting. Sedentary behaviours (SB) are associated with metabolic diseases (e.g., type 2 diabetes mellitus), cancer, musculoskeletal conditions, cardiorespiratory diseases, possibly increasing mortality, especially among individuals with poor socioeconomic status [5, 6].

The World Health Organization has promoted the Global Action Plan on Physical Activity 2018–2030 [1], intending to improve PA by 15% by 2030. The plan includes 20 policy actions to ensure equal opportunities and enable the environment to be physically active, including digital interventions such as mobile apps, remote counselling, and wearable devices (WDs). WDs for physical activity tracking, are electronic non-invasive monitoring devices, mainly in the form of wrist devices that enable the tracking of PA metrics (e.g., the number of steps taken, energy expenditure, time spent sleeping and time spent in different activities levels) [7, 8]. In particular, WDs might serve as a useful tool to be included in broader programmes to increase PA, stress management, physical and mental quality of life, and reduce SB and weight [9, 10].

The global fitness tracker market has been valued at around US$ 40 billion in 2022, up from US$ 36 billion in 2020, and is expected to expand to US$ 46 billion in 2023 globally (https://market.us/report/fitness-tracker-market/#overview). The market value is forecast to reach US$ 187 billion by 2032 (https://market.us/report/fitness-tracker-market/#overview). Increasing market growth has occurred concomitantly with research, as several systematic reviews (SRs) have been published on the efficacy of WDs. To date, contrasting results are reported when activity was measured as SB or light PA [11–13]. One umbrella review systematically analysed secondary studies on the effectiveness of WDs on PA levels [14], retrieving systematic reviews published until April 2021, with WDs as a key intervention. There is an opportunity for an updated umbrella review, encompassing the latest systematic reviews on WDs, either alone or combined with other interventions. Furthermore, the aforementioned recent umbrella review [14] did not investigate SB, providing room for improvement for evaluating the efficacy of WDs, considering that SB is widely studied alongside physical activity. This umbrella review aims to summarise the available SRs regarding the efficacy of WDs use on increased PA levels and reducing SB in adults.

### Research Question

The research question was: does using WDs increase PA levels and reduce SB in adults (aged ≥ 18 years)?

## Methods

We conducted an umbrella review of SRs in accordance with the Cochrane Handbook’s chapter on overviews of reviews and the Joanna Briggs Institute Manual for Evidence Synthesis [15, 16]. We followed the Preferred Reporting Items for Systematic Reviews and Meta-Analyses (PRISMA) [17] for the flow chart and the Preferred Reporting Items for Overviews of Reviews (PRIOR) [18, 19], as reporting checklist (Additional file 1: Supplemental File 1). The review protocol was registered in the International Prospective Register of Systematic Reviews (PROSPERO) database (CRD42022339140). Summary of methods and deviations from the protocol are reported in Additional file 1: Supplemental File 2.

## Eligibility Criteria

### Types of Interventions

We included systematic reviews that investigated the use of WDs to improve PA levels. WDs included devices such as accelerometers, pedometers, Electronic Activity Monitor Systems (EAMSs), or global positioning systems (GPS). We included the use of WDs when it was the only component of the intervention or when it was included in a multi-component intervention. Control groups included active, passive, or no interventions, as originally described by SR authors in their eligibility criteria. Passive interventions were defined as those minimal interventions related to PA (e.g., PA educational booklets, PA and dietary counselling), standard of care (e.g., routine outpatient follow-up, standard medical advice) or wait list assignment. Active interventions were considered the same intervention of the intervention group but delivered without WDs, with WDs but blinded, or with another intervention to promote PA.

### Types of Outcome Measures

The primary outcomes were PA level and SB. Physical activity was measured objectively in terms of the number of steps per day, minutes of moderate to vigorous physical activity (MVPA) per week, and/or any composite measurements (e.g., metabolic equivalent for a task [MET], min/week, intensity, time spent walking), whereas SB was objectively measured by minutes per day.

### Types of Studies

**Fig. 1:**
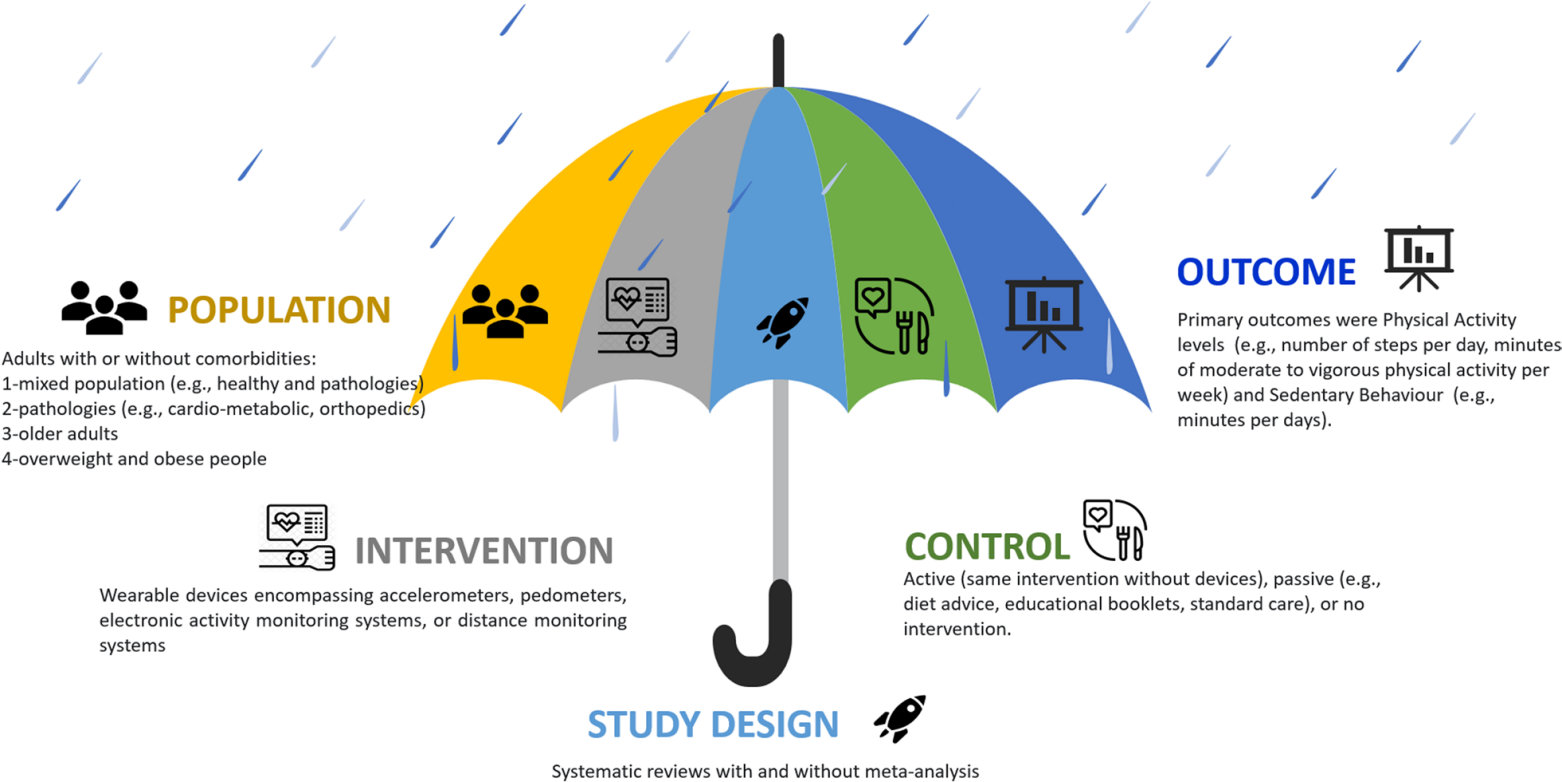
Eligibility criteria

In accordance with Cochrane’s definition, all SRs of primary studies (e.g., randomized controlled trials [RCT]) with or without meta-analysis were included [20]. No restrictions on language and publication date were applied. Figure 1 summarized the eligibility criteria.

### Search Strategy

Two independent authors (JL, CM) launched the search strategy (Additional file 1: Supplemental File 2) on June 10th, 2022, and updated it on February 5th, 2023, to include the most updated evidence through the following databases: PubMed, Cumulative Index to Nursing and Allied Health Literature, Cochrane Database of Systematic Reviews including the Database of Abstracts of Reviews of Effects (DARE). Both free and MeSH (Medical Subject Headings) terms were used. In addition, a free search was also performed through scientific websites (MedRxiv, Rxiv and bioRxiv databases) adapting the search strategy provided for other databases. If a published scientific version was available in a journal article, we prioritized it. We also checked references of included studies to include other potential reviews.

### Study Screening and Selection

Records retrieved were processed through EndNote X8.2 (Clarivate, Philadelphia) to eliminate duplicates and then uploaded onto the Rayyan website [21] for selection. Afterwards, two independent researchers (JL, CM) screened records, applying the eligibility criteria to titles and abstracts. Potential eligible records were retrieved to read the full text and determine the final inclusion. A third author (GR) was consulted to reach a consensus in cases of disagreement between reviewers. We evaluated the agreement in the screening process of full-text by Cohen’s kappa statistics resulting in 0.83 (interquartile range, IQR 0.75 – 0.91), indicating a near-perfect agreement [22].

### Data Collection

Two independent researchers (SG, SB) extracted the data exactly as they were reported from the original SR using a standardised Microsoft® Excel® 2019 MSO spreadsheet. The extracted data included: characteristics of the SRs (title, year of publication, first author, journal, study design, objective, population analysed, outcome studied) and characteristics of the primary studies included in each review (number and typology of studies, population inclusion criteria, intervention, control, brands of WDs). We extracted mean difference (MD) or standardized mean difference (SMD) for quantitative results related to PA, expressed as continuous outcomes.

To summarize the effect estimates and the certainty of the evidence, when additional controls were available, data were extracted on the following a priori-defined list: (1) passive control and (2) other active intervention. Either the shortest available follow-up data or the available measurements reported for the meta-analyses were used since the aim of this umbrella review was to assess immediate effects of receiving an intervention. In cases of missing information, the corresponding authors of SRs were contacted. Disagreements in the data collection process were resolved by either a consensus process or consultation with a third author (GR).

### Data Synthesis

We presented the summary of evidence without re-analysing outcome data. Data were extracted as they were reported in the included SRs (with and without meta-analysis) and then reformatted and presented in text, tables, and figures. We described review characteristics such as eligibility criteria to ensure that SRs are investigating similar clinical questions. We grouped SRs into four categories according to the following population: (a) studies on mixed populations, including SRs on adults in general, healthy adults, or mixed populations of healthy and overweight/obese adults or adults with cardiovascular risk factors; (b) studies on populations with pathologies, e.g., cardiometabolic, pulmonary or orthopaedic diseases; (c) studies on older adults, including SRs on adults over 55, or 60, or 65 years old; or (d) studies on overweight and obese populations.

For SRs without meta-analyses, we calculated and then summarized by plotting the percentage of primary studies that found positive findings over the total number of primary studies reporting the outcome (i.e., statistically significant difference in favour of WD). For SRs with meta-analyses, the lists of the primary studies included in each SR with meta-analyses were collated and cross-referenced in a matrix of evidence tables to ascertain the degree of overlap between SRs for each treatment comparison of PA outcome. The “corrected covered area” (CCA) was calculated to quantify the degree of overlap between reviews at both the outcome and population levels. To interpret the results providing context for clinical implications, we followed the decision tree that Hennessy et al. 2021 [23] proposed. We used a conceptual framework presenting results by outcome and population subgroups [24, 25]. In order to visualize findings for steps per day, minutes of MVPA per week, minutes of SB per day and composite outcomes of PA, we showed in a forest plot the effect size of each meta-analysis without calculating the overall pooled estimate. We then created a visual map of the scientific evidence based on bubble plots to display the information of each review as a bubble according to the direction of effect and Certainty of Evidence (CoE) assessment [26], to quickly examine the concordance or discordance of results [27]. Discordances were explained in the case of SRs with similar PICO questions, including the same trials (i.e., moderate, high, very high overlapping) [24, 25, 28].

### Assessment of Methodological Quality

The methodological quality was assessed using “A MeaSurement Tool to Assess systematic Reviews 2 tool” (AMSTAR 2) [29] by two independent researchers (AT, SB). This tool allows for a reproducible critical evaluation of SRs of RCTs and non-randomized studies of interventions (NRSI) in terms of an overall assessment of the reliability of the results included in the SRs (Additional file 1: Supplemental File 3).

### Certainty of the Evidence

Two independent researchers (SG, SB) used the Grading of Recommendations Assessment, Development and Evaluation (GRADE) approach to evaluate the Certainty of Evidence (CoE) of the SRs, adopting the algorithm from Pollock and colleagues [30] for PA, and separately assessing each population category. In this algorithm, each SR starts with a ranking of high certainty and can be downgraded for severe methodological concerns (Additional file 1: Supplemental File 3) [30].

### Clinical Relevance and Overall Interpretation of PA

We adopted the effect size of the main representative SR (i.e., highest number of participants, most updated and highest methodological quality) assessing patients with mixed populations [31] (e.g., pathologies, older adults, obese or overweight people), as a measure of clinical relevance between WD and controls. Accordingly, we imputed 1,235 daily steps, 48.5 min weekly of MVPA, and 9.9 min daily less of SB, as minimal important difference between group interventions. Clinical relevance was interpreted considering the categories proposed by Man-Son-Hing et al. 2002 (e.g., definite, probable, possible, definitely not) [32].

Subgroup populations order of publication year was plotted, including the effect size of all meta-analyses in mean differences, to give an overall interpretation of steps per day, minutes of MVPA per week and minutes of SB. When effect sizes were reported in SMD, we first searched if back-translations were already reported by SRs; otherwise, we back-translated them using the standard deviation of the control of the RCT with the highest number of participants of each meta-analysis [33]. In Additional file 1: Supplemental File 4, all details for the interpretation of clinical relevance and back-translation are reported.

## Results

### Study Inclusion

**Fig. 2:**
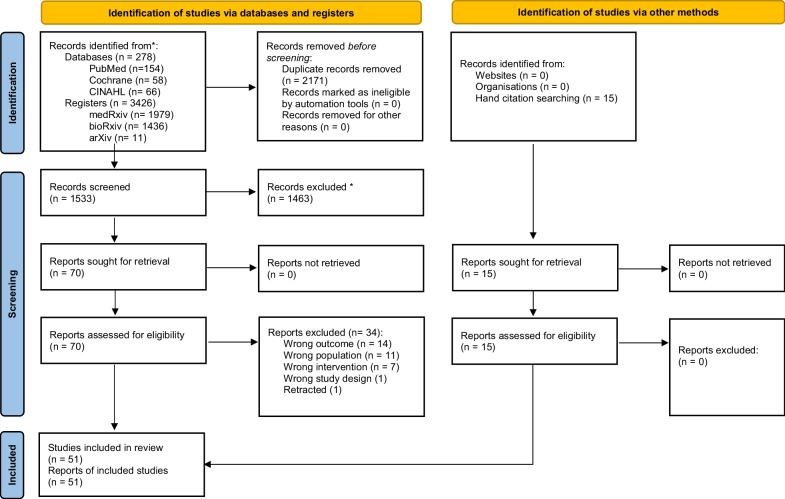
PRISMA 2020 flow diagram for new systematic reviews which included searches of databases, registers and other sources. PRISMA, Preferred Reporting Items for Systematic Reviews and Meta-Analyses; CINAHL, Cumulative Index to Nursing and Allied Health Literature

The search identified 278 publications from databases, 3426 from registers, and 15 through other sources. Of the 85 SRs screened by the full text, 34 were excluded (Additional file 1: Supplemental File 5) and 51 SRs [11, 13, 34–81] were included (Fig. 2).

### Characteristics of Studies Included

The included SRs were published in 37 journals between the dates of 2007 to 2022. Of the 51 included SRs, 74.5% incorporated a meta-analysis. The majority of SRs included only RCTs (80.4%). The characteristics of the SRs are described in Table 1 and details in Additional file 1: Supplemental File 6. The SRs included a median of 17 primary studies and a median of 2,355 participants per SR; across reviews there were 302 unique primary studies. Overall, 22 SRs (43%) included people with pathologies (e.g., orthopaedic, rheumatological, neurological, cardiometabolic, and tumours), 19 (37%) involved a mixed population, five (10%) focused exclusively on obese or overweight people, and five (10%) older adults. Most of the 51 SRs assessed steps per day outcome (*n* = 33), followed by minutes of MVPA per week (*n* = 21), composite outcome (*n* = 17), and minutes SB per day (*n* = 9).

**Table 1:** General characteristics of included systematic reviews (SRs)

Characteristics	SRs with meta-analysis (*n* = 38)	SRs without meta-analysis (*n* = 13)	Overall (*n* = 51)
*Review characteristics*			
Year of publication [*n* (%)]			
2007–2014	3 (8)	1 (8)	4 (8)
2015–2022	35 (92)	12 (92)	47 (92)
Continent of corresponding author of SRs [*n* (%)]			
Europe	16 (42)	4 (31)	20 (39)
North America	6 (16)	5 (38)	11 (22)
South America	0 (0)	0 (0)	0 (0)
Asia	7 (18)	1 (8)	8 (16)
Africa	0 (0)	0 (0)	0 (0)
Oceania	9 (24)	3 (23)	12 (24)
Number of included primary studies [median (IQR)]	12 (18–27)	8 (12–24)	17 (11–26)
Included study design [*n* (%)]			
Randomized controlled trial	38 (100)	3 (23)	41 (80)
Non-randomized controlled trial	0 (0)	0 (0)	0 (0)
Both^a^	0 (0)	10 (77)	10 (20)
*Population characteristics*			
Overall sample size [median (IQR)]^b^	2 401 (1 385–3 636)	1 272 (526–3 374)	2 355 (1 294–3 626)
Mean age [median (IQR)]^c^	56 (49–64)	51 (38–67)	55 (49–64)
Percentage of female participants [median (IQR)]^d^	61 (47–66)	61 (48–66)	61 (50–66)
Type of population [*n* (%)]			
Mixed	15 (39)	4 (31)	19 (37)
Overweight	3 (8)	2 (15)	5 (10)
Older adults	4 (11)	0 (0)	4 (8)
Pathologies	16 (42)	7 (54)	23 (45)
*Treatment characteristics*			
Intervention [*n* (%)]^e^			
Multicomponent	38 (100)	10 (77)	48 (94)
Non multicomponent	0 (0)	1 (8)	1 (2)
*Outcome measurements*^f^			
Physical activity			
Steps per day	22 (58)	11 (85)	33 (65)
Moderate to vigorous physical activity(MVPA)	14 (37)	7 (54)	21 (41)
Composite	16 (42)	2 (15)	17 (33)
Sedentary behaviour (SB)	6 (16)	3 (23)	9 (18)

Steps per day are measured in number of steps, moderate to vigorous physical activity (MVPA) and sedentary behaviour (SB) are measured in minutes per week and day, respectively, composite measurements are measured with standardized mean difference (SMD) as standardized measure of physical activity (e.g., metabolic equivalent for task (MET), min/week, intensity, time spent walking)

^a^E.g., controlled studies, prospective and observational studies

^b^Sample size was not reported in 2 SR + MA and 4 SRs

^c^Mean age was not reported in 5 SRs + MA and 6 SRs

^d^The percentage of female participants was not reported in 13 SRs + MA and 6 SRs

^e^Intervention adopted was not reported in 2 SRs

^f^Some SRs assessed more than one outcome

In 48 SRs (94%), there were multi-component interventions, whereas in two SRs, data were unavailable. Overall, in the SRs with multi-component intervention, the intervention was a combination of WDs as the main component of the intervention, or as part of a multimodal intervention consisting of various elements (e.g., use of a wearable device and a diary to record step count with feedback from a facilitator, or use of the wearable device and telephone support). Across the 48 SRs, the most frequent brands of WDs were Fitbit (*n* = 21), Jawbone (*n* = 12 SRs), and Polar (*n* = 11 SRs)-no data on brands of worn devices were provided in four SRs.

### Methodological Quality

**Fig. 3:**
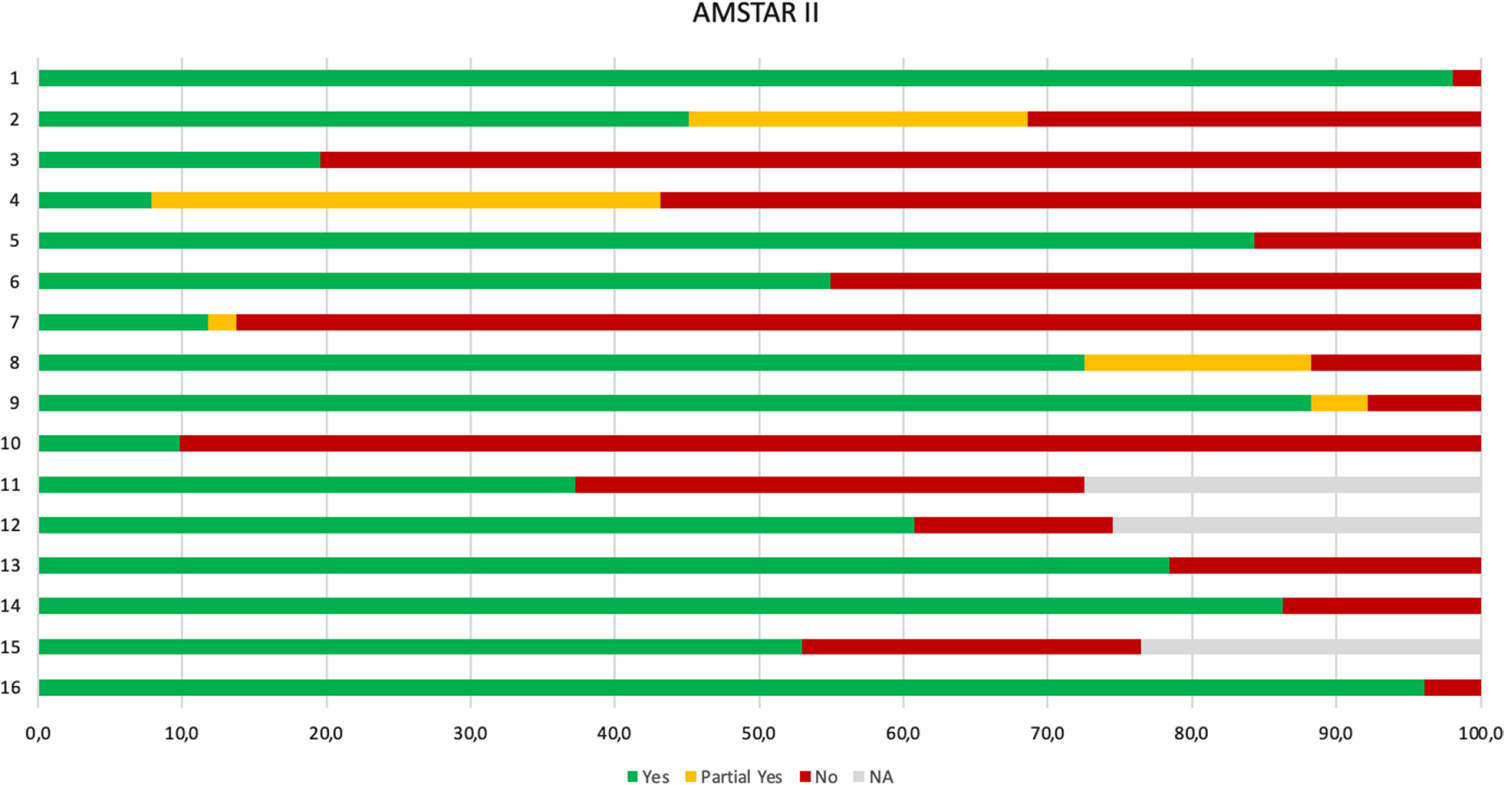
Methodological quality of the 51 SRs according to the 16 items of AMSTAR 2. Item 1 (Did the research questions and inclusion criteria for the review include the components of PICO?); 2: item 2 (Did the report of the review contain an explicit statement that the review methods were established prior to the conduct of the review and did the report justify any significant deviations from the protocol?); 3: item 3 (Did the review authors explain their selection of the study designs for inclusion in the review?); 4: item 4 (Did the review authors use a comprehensive literature search strategy?) 5: item 5 (Did the review authors perform study selection in duplicate?); 6: item 6 (Did the review authors perform data extraction in duplicate?); 7: item 7 (Did the review authors provide a list of excluded studies and justify the exclusions?); 8: item 8 (Did the review authors describe the included studies in adequate detail?); 9: item 9 (Did the review authors use a satisfactory technique for assessing the risk of bias (RoB) in individual studies that were included in the review?); 10: item 10 (Did the review authors report on the sources of funding for the studies included in the review?); 11: item 11 (If meta-analysis was performed, did the review authors use appropriate methods for statistical combination of results?); 12: item 12 (If meta-analysis was performed, did the review authors assess the potential impact of RoB in individual studies on the results of the meta-analysis or other evidence synthesis?); 13: item 13 (Did the review authors account for RoB in primary studies when interpreting/discussing the results of the review?); 14: item 14 (Did the review authors provide a satisfactory explanation for, and discussion of, any heterogeneity observed in the results of the review?); 15: item 15 (If they performed quantitative synthesis did the review authors carry out an adequate investigation of publication bias (small study bias) and discuss its likely impact on the results of the review?); 16: item 16 (Did the review authors report any potential sources of conflict of interest, including any funding they received for conducting the review?)

The evaluation with the AMSTAR 2 checklist outlines that confidence in the results of 51 SRs (72.5%) was rated as ‘critically low confidence’, 11 (21.6%) as ‘low confidence’, and three (5.9%) as ‘moderate confidence’. The primary critical weaknesses corresponded to not providing a list of excluded studies with a justification of the reasons (*n* = 44), not using a comprehensive literature search strategy (*n* = 29), and not justifying the choice of meta-analysis as an appropriate tool for the statistical combination of results (*n* = 19). The most frequent flaws of non-critical weaknesses were not reporting the sources of funding of the studies included in the SRs (*n* = 46), not justifying the choice of the design of the studies included in the SRs (*n* = 41), and not performing the extraction data by at least two independent authors (*n* = 3) (Fig. 3). AMSTAR 2 assessments for each SR are reported in Additional file 1: Supplemental File 7.

### Systematic Reviews Without Meta-Analysis

We found sparse effects that favoured WD from primary studies included in the 13 SRs without meta-analysis. The most reported outcomes were PA (generically and inconsistently defined) and SB. On average, the proportion of trials reporting statistically significant results were 56% (95%CI 0.23%-0.81%) and 32% (95%CI 0.11%-0.69%), respectively (Additional file 1: Supplemental File 8).

### Systematic Reviews with Meta-Analysis

#### Overlapping

**Fig. 4:**
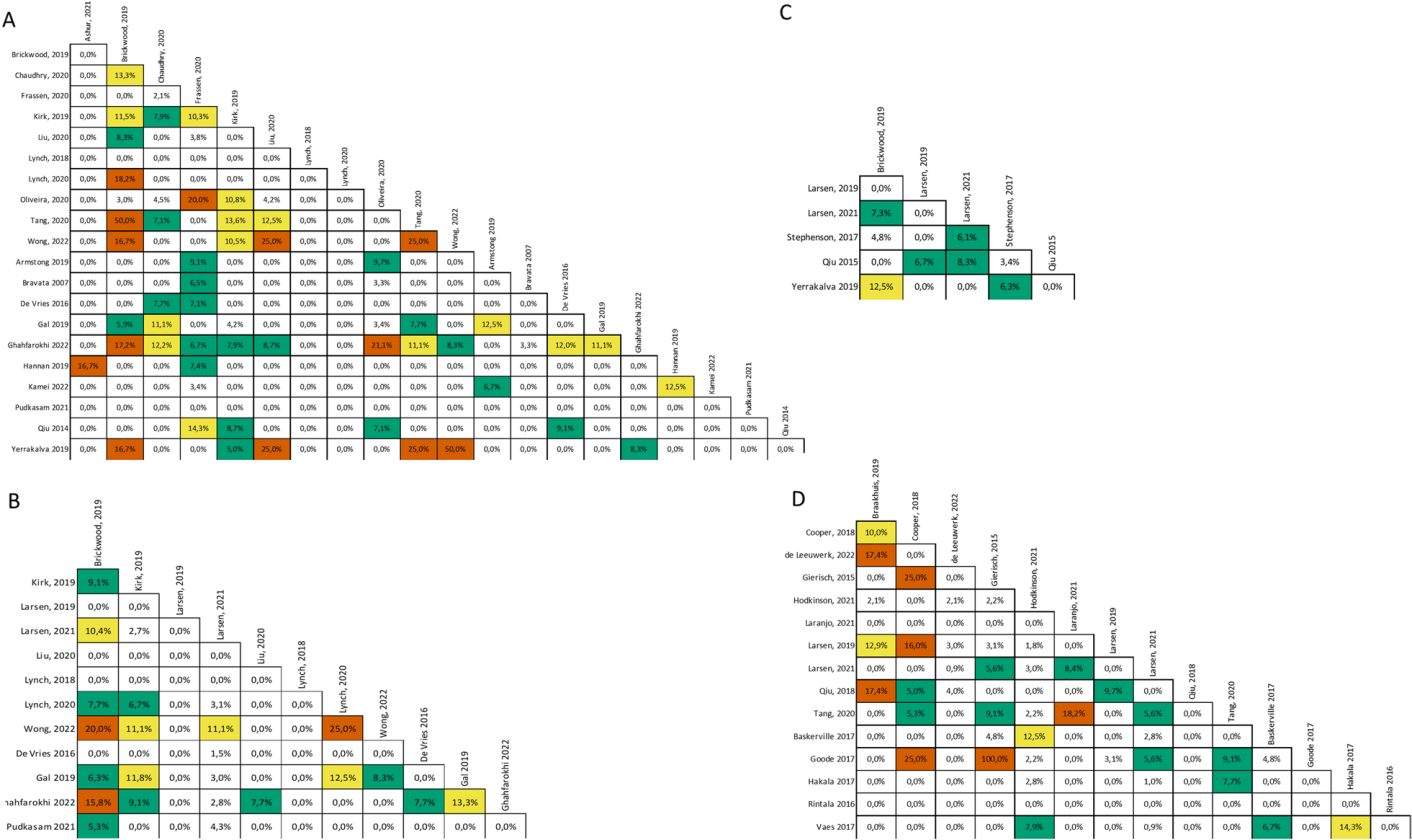
Overlapping of primary studies across SRs in **A** step per day, **B** MVPA, **C** SB, and **D** composite measurements. White, slight overlap (< 5%), green moderate overlap (5% to < 10%), yellow high overlap (10% to < 15%), orange very high overlap (≥ 15%). MVPA, moderate to vigorous physical activity; SB, sedentary behaviour. Step per day is measured in number of steps, MVPA and SB are measured in minutes, composite measurements is measured in standardized mean difference (SMD) as standardized measure of physical activity (e.g., metabolic equivalent for task (MET), min/week, intensity, time spent walking)

Of the 57 meta-analyses included in the 38 SRs, four meta-analyses from two SRs [43, 71] were not considered, because of unclear reporting of primary studies and measure of effect. At the outcome level, we found a slight overlap of citation of primary studies with a CCA of 3.87% for steps per day, 3.12% for minutes of MVPA per week, 4.06% for minutes of SB per day and 2.68% for composite measurements (Fig. 4). Similar overlap was reported in subgroup analysis for population, except for steps per day in obese/overweight people and mixed populations, where we found moderate overlap. Overlap was reported in the subgroup of the population at the outcome level (Additional file 1: Supplemental File 9).

#### Efficacy Results

Of the 53 meta-analyses considered for the analysis, most favored intervention using WDs with low-to-moderate certainty of evidence (CoE) (*n* = 43, 81.1%). The remaining 10 found no differences between WD and comparators. In Additional file 1: Supplemental File 10, we report all the effect sizes with CoE assessments and AMSTAR 2 ratings, whereas in Additional file 1: Supplemental File 11, we report bubble plots linking CoE with the direction of effect overall and stratified for each outcome, underlining concordances and discordances in the direction of the effects.

#### Physical Activity

##### Steps Per Day

Seventeen out of 21 meta-analyses (median 679 [IQR 298.7–1474.5] participants) favoured interventions using WDs with low-to-moderate CoE, whereas four reported no differences. At the population level, considering meta-analyses with moderate CCA, we found concordance on the superiority of WDs in all meta-analyses on mixed (*n* = 6, CCA moderate 6.98%) and obese/overweight (*n* = 3, CCA moderate 9.62%) populations. In contrast, discordance was found in older adults (*n* = 2 favour intervention, *n* = 1 no differences) and people with pathologies (*n* = 6 favour interventions, *n* = 3 no differences). These discordant meta-analyses presented a slight overlap in conditions (older adults 3.85%, pathologies 3.13%).

##### Minutes of Moderate to Vigorous Physical Activity Per Week

Eleven out of 12 meta-analyses (median 1,206 [IQR 519–1665] participants) favoured interventions using WDs with low-to-moderate CoE, whereas one found no differences. At the population level, we found concordance on the superiority of WDs in all meta-analyses on obese/overweight (*n* = 3) and people with pathologies (*n* = 3), whereas discordances were found in a mixed population (*n* = 4 favour intervention, 1 no difference). The only meta-analysis on older adults found superiority of WDs over controls. The overlap at the population level was slight (mixed population 3.48%, people with pathologies 0%, obese/overweight people 2.5%).

##### Composite Measurements

Thirteen out of 14 meta-analyses (median = 1356 [IQR 867–1435] participants) favoured interventions using WDs with low-to-moderate CoE, whereas one found no difference between groups. At the population level, we found concordances in the superiority of WDs in all the meta-analyses on mixed populations (*n* = 7) and people with pathologies (*n* = 6). The overlap at the population level was slight (mixed population 4.56%, people with pathologies 3.06%). The only meta-analysis on older adults found no differences between groups. No meta-analyses on obese/overweight people were reported by SRs.

#### Minutes of Sedentary Behaviour Per Day

Two out of six meta-analyses (median = 1189 [IQR 288.5–2797] participants) favoured interventions using WDs with low-to-moderate CoE, whereas four found no differences. At the population level, we found discordances in a mixed population (*n* = 2 favour intervention, *n* = 3 no difference) with a slight overlap (4.3%). The only meta-analysis on the older adults found no differences between groups. No meta-analysis on obese/overweight people and people with pathologies were found across SRs.

#### Certainty of Evidence

Of the 53 meta-analyses, 29 were rated as moderate CoE (*n* = 12 steps per day, *n* = 7 MPVA, *n* = 5 SB, *n* = 5 composite measurements), 21 as low CoE (*n* = 8 steps per day, *n* = 3 MPVA, *n* = 9 composite measurements, *n* = 1 SB), and for three, the overall assessment was not possible (*n* = 1 steps per day, *n* = 2 MPVA). Reasons for downgrading were mainly due to serious (*n* = 19) and very serious (*n* = 12) limitations of methodological quality of SRs, inconsistency (I^2^ > 75%) (*n* = 23), and risk of bias at the trial level (*n* = 22). Most meta-analyses (*n* = 47) involved more than 200 participants, indicating precise effect sizes (Additional file 1: Supplemental File 10).

#### Clinical Relevance and Overall Interpretation of PA and SB

**Fig. 5:**
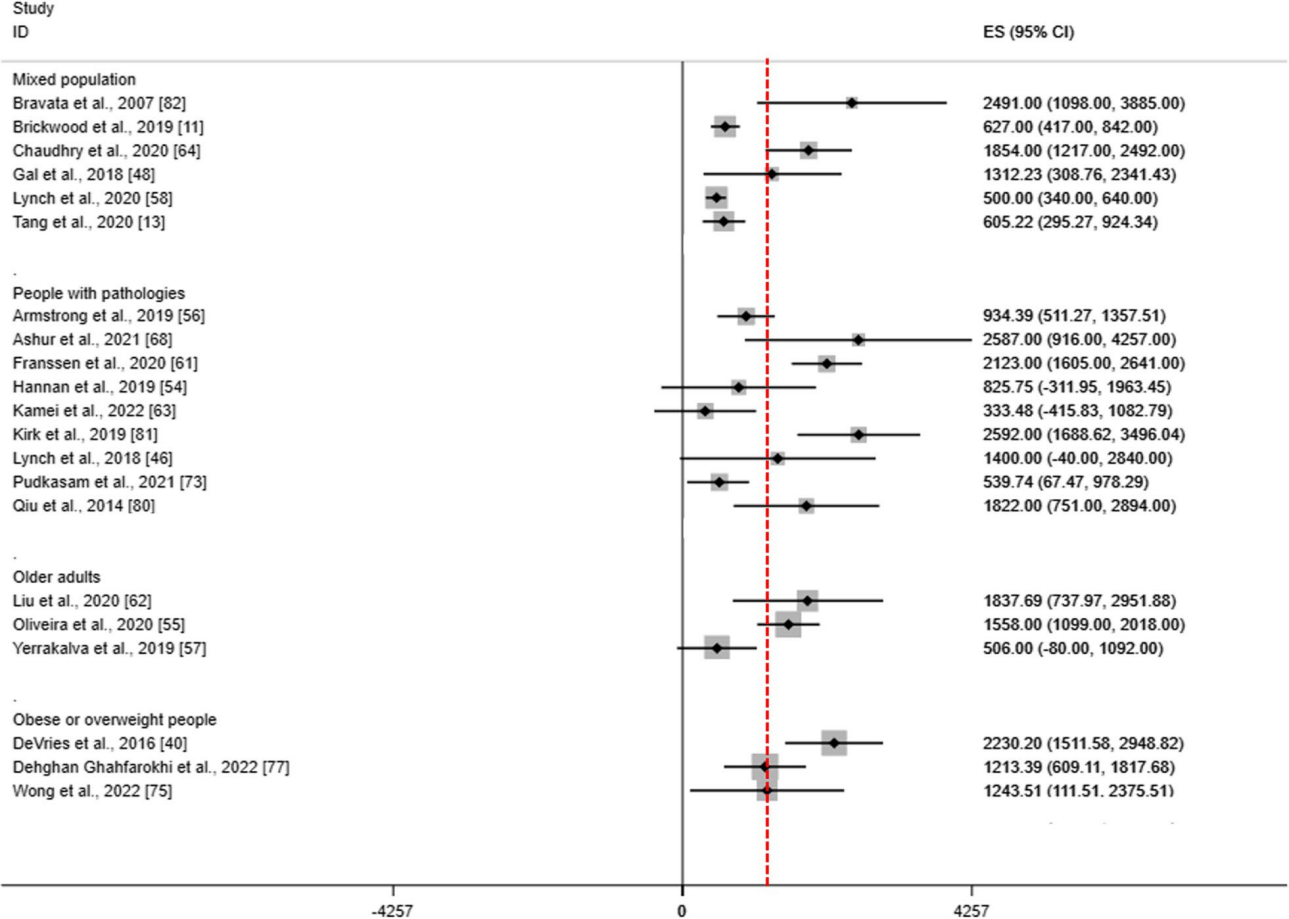
Summary of results of steps per day. Red line refers to the clinical relevance (Larsen 2021 [31]). Step per day is measured in number of steps per day. When effect sizes were reported in SMD, back-translation were obtained using the standard deviation of the control group of the RCT with the highest number of participants of each meta-analysis

**Fig. 6:**
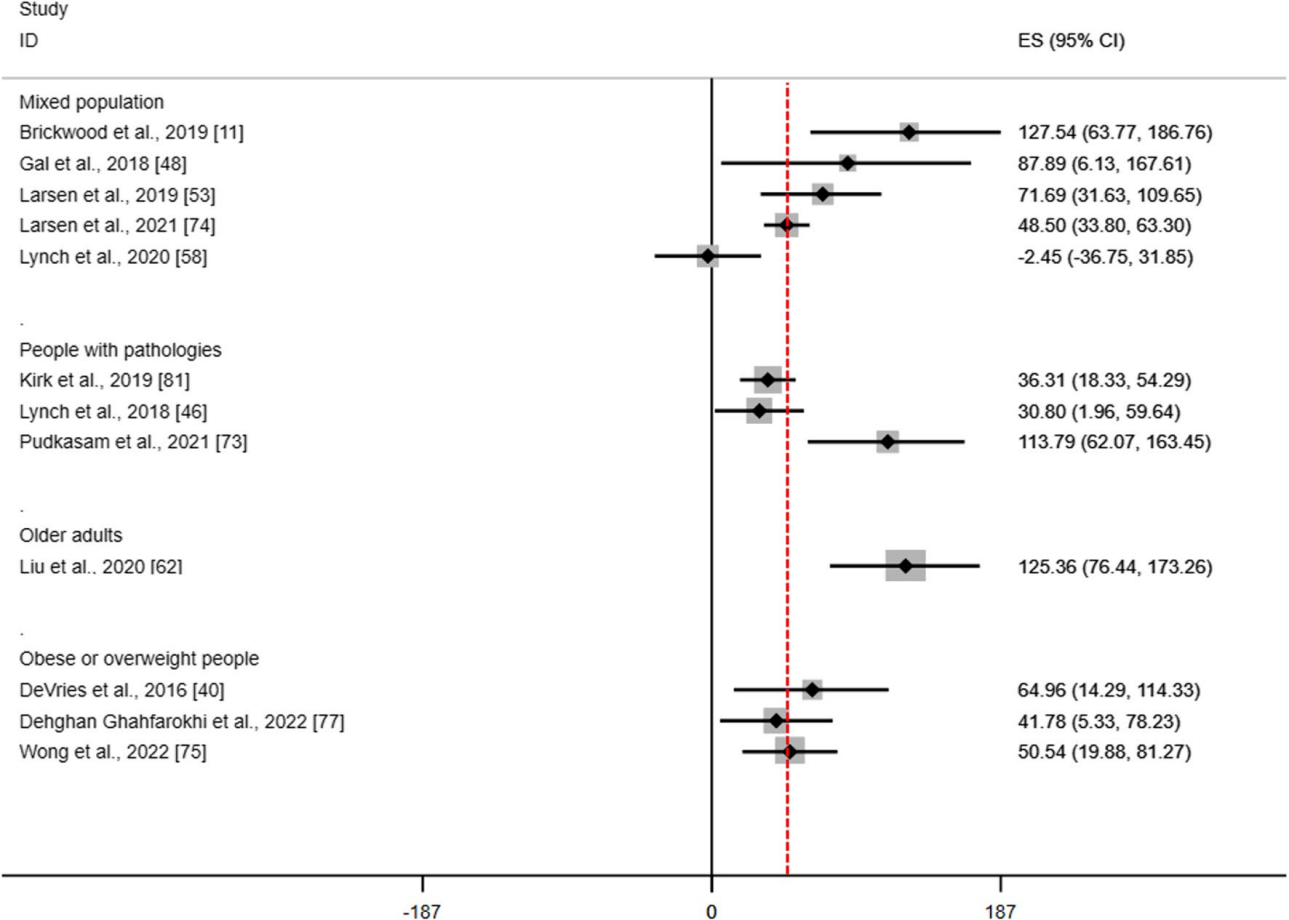
Summary of results for minutes of MVPA per week. Red line refers to the clinical relevance (Larsen 2021 [31]). MVPA, moderate to vigorous physical activity. MVPA is measured in minutes per week. When effect sizes were reported in SMD, back-translation were obtained using the standard deviation of the control group of the RCT with the highest number of participants of each meta-analysis

In Figs. 5 and 6, we plotted MDs of all meta-analyses for steps per day and MVPA according to population categories, whereas in Additional file 1: Supplemental File 12 we plotted SB and composite measurements. Overall, WDs may increase PA by a median of 1,312.23 (IQR 627–1854) steps per day and 57.8 (IQR 37.7 to 107.3) minutes of MVPA per week and may reduce minutes of SB by a median of -27.76 (IQR -41.28 to 9.9) per day. Clinical relevance was found definitive for 15% and ‘probable to possible’ for 48% of SRs for steps per day; definitive for 25%, and ‘probable to possible’ for 67% of SRs for minutes of MVPA per week; and definitive for 16.7% and probable for 33% of SRs for minutes of SB per day (Additional file 1: Supplemental File 12).

## Discussion

### Main Findings

Overall, our results were consistent across different PA outcomes (steps per day, MVPA, and PA as composite outcome) in almost all the SRs, while few discordances in SB were found. With low to moderate CoE and ‘possible to definitive clinical relevance’, using WDs may increase PA by a median of 1,312.23 (IQR 627–1854) steps per day, and a median of 57.8 (IQR 37.7 to 107.3) minutes per week of MVPA compared to passive controls. Sparse results were found for minutes of SB per day (two out of six SRs favoured intervention; median of -27.76 min [IQR -41.28 to 9.9]) with imprecise confidence intervals (i.e., reaching clinical relevance but not statistically significantly different). When we explored the results across different population, SRs reported consistent results on the efficacy of WDs on PA in mixed population (steps per day and composite outcomes) in populations with pathologies (MVPA and composite outcomes) and for obese or overweight populations (steps per day and MVPA). Among older adults, results were inconsistent across outcomes (discordance on steps per day, positive findings on MVPA, no difference on SB), and emerged from small samples in few SRs (< 200 participants for steps per day and MVPA).

Generally, the results are comparable to those detected by a previous umbrella review which reported that the use of activity trackers improved PA except for older adults [14]. However, the SB outcome was not covered by this review [14]. In our umbrella review, we found some uncertainty for PA in pathologies and older adults subgroups and for SB in mixed and older adults subgroups. Considering the subgroup with pathologies (*n* = 23 SRs), we found poorly informative SRs with large confidence intervals in quantitative meta-analyses (*n* = 16 SRs) and variability of effects in SRs with qualitative synthesis in approximately one-third of all SRs (*n* = 7 SRs). On one hand, some populations, such as orthopaedic patients (osteoarthritis, low back pain), may benefit from physical activity, in terms of locomotor function, balance and strength, whereas other frail populations, with reduced airflow and cardiac capacity, could encounter barriers when trying to increase PA [82]. However, a recently published SR [83], which included 38 studies on a population with chronic airway diseases, confirmed the positive results in improving PA when using WDs. Considering the subgroup of older adults, we found different effects compared to the previous umbrella review of Ferguson et al. [14], which found positive effects on steps per day in this population. The difference might be explained by the absence of a SR that we included [57]. This SR [57] did not reach statistical significance for steps per day, contributing to our inconsistent findings. For older adults, increasing their PA levels through the use of WDs might be hampered by difficulty using new technologies, such as activity monitors [50]. This point aligns with Franssen and colleagues [61], who reported that younger peoples’ use of wearable trackers was associated with a significantly greater increase in PA. Evidence suggests that engagement in eHealth interventions is reduced among older adults with a lower level of formal education, limited computer experience, and poorer cognition [84]. Strategies to overcome this barrier could consist of introducing scheduled follow-up assessments by meetings and telephone consultations [85], in light of evidence of poor communication with health professionals and lack of feedback and human support as hindrances to the acceptance and usability of digital technologies in older adults [86, 87].

All SRs with meta-analysis included multicomponent interventions in which WD was the tool used in implementing the activity-based approach. After sub-analysing the types of interventions investigated among SRs, we found that WDs could be more effective when associated with feedback, coaching, or motivational interventions, rather than as a stand-alone intervention, even across populations with pathologies. For example, Laranjo et al. [65] have demonstrated that the intervention might be more effective if it includes text conveying motivational messages or personalisation (e.g., personalized goal setting, contents and feedback). Furthermore, in keeping with the prior report, interventions might be more effective if based on theories of self-regulation and if interactive functions are adopted to engage users in behaviour change [88]. For example, artificial intelligence chatbots show great promise in promoting healthy lifestyles and physical activity [89], based on several features such as understanding user background, establishing persuasive conversations, and providing input according to behavioural outcomes [90].

Optimal follow-up timing was unclear in most SRs, suggesting a gap in research regarding the most effective length of intervention. The relevance of follow-up length may be related to poor long-term behavioural changes needed with use of WDs, possibly due to the waning initial novelty of WD interventions [91]. More than half of the people who buy a wearable activity device stop using it, of which 1/3 stop within the first months [38].

### Research and Clinical Practice Implications

Our findings suggest that using WDs to promote PA may be effective in populations with and without diseases. However, careful attention should be paid before transferring these results into clinical practice. The CoE was low in less than half of the meta-analysis, meaning that the true effect might be different from the estimated effect. Second, inconsistent results emerged regarding the efficacy of WDs for PA in pathologies and older adults’ subgroups and for SB in mixed and older adults’ subgroups. Third, the follow-up timing was unclear, thus not providing information on the best length of intervention. Fourth, the age of the samples included in the SRs was not always reported, limiting the comparison across SRs. Fifth, the SRs included different types of WDs: each device may have a different accuracy in measuring PA (sensitivity or specificity), which could affect the overall estimation of the effects. Finally, the Hawthorne effect may have influenced the results of the studies analysed by the SRs. For instance, the awareness of being part of a physical activity study could have prompted participants in the control group to increase their activity levels. [53].

As implications for research, the efficacy of WDs should be further investigated, especially in the long-term and on SB. Future studies are needed to investigate the effectiveness of WDs among older adults and if additional components (e.g., telephone follow-up) might improve WDs' effect in this population. It would be helpful to investigate more in-depth in all populations if and which additional intervention components can increase the effect size with repercussions on PA.

Despite these issues, our findings are important for healthcare professionals, who may consider WDs to improve people's health and well-being. For example, there are clear dose–response associations between increasing step counts and decreasing mortality, with 1,000 more steps per day associated with a 15% lower risk in older men [92] and 6% in younger men and women [93]. In addition, the NAVIGATOR study [94], which includes 9,000 individuals with high cardiovascular risk or impaired glucose tolerance, showed that for every increase of 2000 steps per day, the risk of developing cardiovascular problems decreased by 10%, the risk of developing type 2 diabetes mellitus (T2DM) by 5.5%, and the metabolic syndrome risk score was reduced by 0.29 [94–96]. A recent large cohort study on 81,717 participants showed that an increase of 20 min in daily MVPA is associated with a reduction in hospitalization ranging from 3.8% for colon polyps, 14.1% for pneumonia, 19.8% for gallbladder disease, 22.7% for urinary tract infection, and 23% for diabetes [97]

### Strengths and Limitations

To our knowledge, this is the most comprehensive umbrella review investigating the effectiveness of WDs on PA levels. We set a population limit for adults aged over 18 years, allowing a wide breadth of assessment. A comprehensive hand search detected additional reports for inclusion. We considered different outcomes when assessing PA that allowed us to detect most of the sensitive measures for evaluating the efficacy of WDs. We analysed overlap among SRs, both at the outcome and population level, discovering a slight overlap among most comparisons. This further corroborates our conclusions on the potential benefits of using WDs to improve PA.

There are limitations to this umbrella review. Although the decision to include a wide range of populations may provide a broader understanding of the role of WDs, the clinical heterogeneity included in the SRs (e.g., age, type of intervention and comparison, how to use the device, outcome measures), suggests difficulty in comparison and summarization of results, and produces statistical heterogeneity [98]. In addition, we did not investigate the specific effect of a WD in the presence of multi-component interventions. We adopted the compared interventions as they were reported by the original SR authors, relying on their eligibility criteria and analyses. We cannot exclude bias in the conduct of SRs, although we attempted to reduce this by assessing the methodological quality of SRs. However, we cannot rule out that these factors may have affected the significance of the results.

## Conclusions

Our results indicate that the adoption of WDs represents a beneficial approach to enhance physical activity across diverse population groups, although there is some level of uncertainty, particularly in the subgroups of individuals with pathologies and older adults, as well as in the case of sedentary behaviour within mixed and older adults' subgroups. Further research is required to enhance the precision of the effects within all population subgroups.

### Supplementary Information

**Additional file 1**. Supplementary Materials.

## Data Availability

The full dataset is freely available online in OSF (https://osf.io/udptm/), a secure online repository for research data.

## Abbreviations

AMSTAR 2: Assessment of multiple systematic reviews, version 2
CCA: Corrected covered area
CoE: Certainty of evidence
COPD: Chronic obstructive pulmonary disease
DARE: Database of abstracts of reviews of effects
EAMSs: Electronic activity monitor systems
GPS: Global positioning system
GRADE: Grading of recommendations, assessment, development, and evaluations
IQR: InterQuartile range
MD: Mean difference
MeSH: Medical subject headings
MET: Metabolic equivalent
MVPA: Moderate to vigorous physical activity
NRSI: Non-randomized studies of interventions
PA: Physical activity
PRIOR: Overviews of reviews
PRISMA: Preferred reporting items for systematic reviews and meta-analyses
PROSPERO: Prospective register of systematic reviews
RCT: Randomized controlled trials
SB: Sedentary behaviour
SMD: Standardized mean difference
SR: Systematic reviews
WD: Wearable devices
