# Supplementary material for: Wearable Devices to Improve Physical Activity and Reduce Sedentary Behaviour: An Umbrella Review
Source: Sports Med Open. 2024 Jan 14;10:9. doi: 10.1186/s40798-024-00678-9 (PMC10788327; doi:10.1186/s40798-024-00678-9)
Supplement: Supplementary file 1 — Additional file 1. Supplementary Materials. [file 40798_2024_678_MOESM1_ESM.docx]

**Electronic Supplementary Material**

**Wearable devices to Improve Physical Activity: an umbrella review**

# Cover page

**Authors:**

Jessica Longhini^1 §^, Chiara Marzaro^2 §^, Silvia Bargeri^3 §^, Alvisa Palese^1^, Andrea Dell’Isola^4^, Andrea Turolla^5,6^, Paolo Pillastrini^5,6^, Simone Battista^4,7^, Greta Castellini^3^, Chad Cook^8^, Silvia Gianola^3 #*^, Giacomo Rossettini^9#^

^1^ Department of Medical Sciences, University of Udine, Udine, Italy.

^2^ Independent researcher, Vicenza, Italy.

^3^ Unit of Clinical Epidemiology, IRCCS Istituto Ortopedico Galeazzi, Milan, Italy.

^4^ Department of Clinical Sciences Lund, Clinical Epidemiology Unit, Orthopedics, Lund University, Lund, Sweden.

^5^ Department of Biomedical and Neuromotor Sciences (DIBINEM), Alma Mater Studiorum Università di Bologna, Bologna, Italy.

^6^ Division of Occupational Medicine, IRCCS Policlinico Sant'Orsola-Malpighi, Bologna, Italy.

^7^ Department of Neurosciences, Rehabilitation, Ophthalmology, Genetics, Maternal and Child Health, University of Genoa, Campus of Savona, Savona, Italy.

^8^ Department of Orthopaedics, Division of Physical Therapy, Duke University, Durham, North Carolina, USA.

^9^ School of Physiotherapy, University of Verona, Verona, Italy

^*^ Corresponding author [silvia.gianola@grupposandonato.it](mailto:silvia.gianola@grupposandonato.it)

^§^ These authors contributed equally, sharing the first authorship

^#^ These authors contributed equally, sharing the last authorship

Index

[Cover page 1](#_Toc148623076)

[Supplementary File 1. Preferred reporting items for overviews of reviews statement (PRIOR) 4](#_Toc148623077)

[Supplementary File 2. EMethods 7](#_Toc148623078)

[Study design and methods 7](#_Toc148623079)

[Figure S1. Methods 7](#_Toc148623080)

[Patient and public involvement 7](#_Toc148623081)

[Equity, diversity, and inclusion statement 7](#_Toc148623082)

[Difference between protocol and publication 7](#_Toc148623083)

[Search strategy 8](#_Toc148623084)

[Supplementary File 3. Methodological Quality and Certainty of the evidence 11](#_Toc148623085)

[Methodological Quality and Certainty of Evidence criteria 11](#_Toc148623086)

[Supplementary File 4. Clinical relevance 13](#_Toc148623087)

[Figure S1. Framework for interpretation of clinical relevance 13](#_Toc148623088)

[Supplementary File 5. Excluded systematic reviews according to eligibility criteria 14](#_Toc148623089)

[Supplementary File 6. General characteristics 17](#_Toc148623090)

[Table S1. Characteristics of included systematic reviews 17](#_Toc148623091)

[Table S2. Type of outcomes assessed in SR with meta-analysis 27](#_Toc148623092)

[Table S3. Type of outcomes assessed in SR without meta-analysis 29](#_Toc148623093)

[Supplementary File 7. Methodological quality in systematic reviews in accordance with AMSTAR 2 30](#_Toc148623094)

[Table S1. AMSTAR 2 of each included SR 31](#_Toc148623095)

[Figure S1. Methodological quality of the 51 SR according to the 16 items of AMSTAR 2 34](#_Toc148623096)

[Supplementary File 8. Systematic Review without Meta-Analysis 35](#_Toc148623097)

[Table S1. Findings in Systematic Review without Meta-Analysis 35](#_Toc148623098)

[Figure S1. Proportion of trials with positive results in SRs without meta-analysis 36](#_Toc148623099)

[Supplementary File 9. Overlapping Corrected Covered Area (CCA) 37](#_Toc148623100)

[Descriptive characteristics of overlapping 37](#_Toc148623101)

[Figure S1. Physical activity as Steps per day 38](#_Toc148623102)

[Figure S2. Physical activity as moderate to Vigorous Physical Activity (MVPA) 42](#_Toc148623103)

[Figure S3.Physical activity as composite measurements 45](#_Toc148623104)

[Figure S4. Sedentary Behaviour 47](#_Toc148623105)

[Supplementary File 10. Meta-analyses effects sizes with certainty of evidence and AMSTAR 2 ratings 48](#_Toc148623106)

[Table S1. Physical activity as Steps per day 48](#_Toc148623107)

[Table S2. Physical activity as moderate to Vigorous Physical Activity (MVPA) 50](#_Toc148623108)

[Table S3. Physical activity as composite measurements 51](#_Toc148623109)

[Table S4. Sedentary behaviour 52](#_Toc148623110)

[Supplementary File 11. Bubble plots linking Certainty of Evidence with direction of effect 53](#_Toc148623111)

[Figure S1. Map of evidence 53](#_Toc148623112)

[Figure S2. Physical activity as steps per day 54](#_Toc148623113)

[Figure S3. Physical activity as Moderate to Vigorous Physical Activity (MPVA) 55](#_Toc148623114)

[Figure S4. Physical activity as composite outcome 56](#_Toc148623115)

[Figure S5. Sedentary behavior 57](#_Toc148623116)

[Supplementary File 12. Clinical relevance 58](#_Toc148623117)

[Figure S1. Plotting Measure Effects – Sedentary behaviour 58](#_Toc148623118)

[Figure S2. Plotting Measure Effects – Composite measurements 59](#_Toc148623119)

[Table S1. Clinical relevance assessment 60](#_Toc148623120)

[References 61](#_Toc148623121)

# Supplementary File 1. Preferred reporting items for overviews of reviews statement (PRIOR)

| Section topic | Item No | Item | Location where item is reported |
| --- | --- | --- | --- |
| Title | | |  |
| Title | 1 | Identify the report as an overview of reviews. | Pag. 1 |
| Abstract | | |  |
| Abstract | 2 | Provide a comprehensive and accurate summary of the purpose, methods, and results of the overview of reviews. | Pag. 3 |
| Introduction | | | Pag.4 |
| Rationale | 3 | Describe the rationale for conducting the overview of reviews in the context of existing knowledge. | Pag. 4 |
| Objectives | 4 | Provide an explicit statement of the objective(s) or question(s) addressed by the overview of reviews. | Pag. 5 |
| Methods | | |  |
| Eligibility criteria | 5a | Specify the inclusion and exclusion criteria for the overview of reviews. If supplemental primary studies were included, this should be stated, with a rationale. | Pag. 5 |
|  | 5b | Specify the definition of “systematic review” as used in the inclusion criteria for the overview of reviews. | Pag. 6 |
| Information sources | 6 | Specify all databases, registers, websites, organisations, reference lists, and other sources searched or consulted to identify systematic reviews and supplemental primary studies (if included). Specify the date when each source was last searched or consulted. | Pag. 6, Figure 1 |
| Search strategy | 7 | Present the full search strategies for all databases, registers and websites, such that they could be reproduced. Describe any search filters and limits applied. | Figure 1, Supplementary File 2 |
| Selection process | 8a | Describe the methods used to decide whether a systematic review or supplemental primary study (if included) met the inclusion criteria of the overview of reviews. | Pag. 6 |
|  | 8b | Describe how overlap in the populations, interventions, comparators, and/or outcomes of systematic reviews was identified and managed during study selection. | Pag. 6 |
| Data collection process | 9a | Describe the methods used to collect data from reports. | Pag. 6 |
|  | 9b | If applicable, describe the methods used to identify and manage primary study overlap at the level of the comparison and outcome during data collection. For each outcome, specify the method used to illustrate and/or quantify the degree of primary study overlap across systematic reviews. | Pag. 6-7 |
|  | 9c | If applicable, specify the methods used to manage discrepant data across systematic reviews during data collection. | Pag. 6-7 |
| Data items | 10 | List and define all variables and outcomes for which data were sought. Describe any assumptions made and/or measures taken to identify and clarify missing or unclear information. | Pag. 5 |
| Risk of bias assessment | 11a | Describe the methods used to assess risk of bias or methodological quality of the included systematic reviews. | Pag. 8 |
|  | 11b | Describe the methods used to collect data on (from the systematic reviews) and/or assess the risk of bias of the primary studies included in the systematic reviews. Provide a justification for instances where flawed, incomplete, or missing assessments are identified but not reassessed. | Pag. 8 |
|  | 11c | Describe the methods used to assess the risk of bias of supplemental primary studies (if included). | Not applicable |
| Synthesis methods | 12a | Describe the methods used to summarise or synthesise results and provide a rationale for the choice(s). | Pag. 7 |
|  | 12b | Describe any methods used to explore possible causes of heterogeneity among results. | Pag. 7 |
|  | 12c | Describe any sensitivity analyses conducted to assess the robustness of the synthesised results. | Not applicable |
| Reporting bias assessment | 13 | Describe the methods used to collect data on (from the systematic reviews) and/or assess the risk of bias due to missing results in a summary or synthesis (arising from reporting biases at the levels of the systematic reviews, primary studies, and supplemental primary studies, if included). | Pag. 8 |
| Certainty assessment | 14 | Describe the methods used to collect data on (from the systematic reviews) and/or assess certainty (or confidence) in the body of evidence for an outcome. | Pag. 8 |
| Results | | |  |
| Systematic review and supplemental primary study selection | 15a | Describe the results of the search and selection process, including the number of records screened, assessed for eligibility, and included in the overview of reviews, ideally with a flow diagram. | Pag. 9, Figure 1 |
|  | 15b | Provide a list of studies that might appear to meet the inclusion criteria, but were excluded, with the main reason for exclusion. | Supplementary File 5 |
| Characteristics of systematic reviews and supplemental primary studies | 16 | Cite each included systematic review and supplemental primary study (if included) and present its characteristics. | Pag. 9 |
| Primary study overlap | 17 | Describe the extent of primary study overlap across the included systematic reviews. | Pag. 11 |
| Risk of bias in systematic reviews, primary studies, and supplemental primary studies | 18a | Present assessments of risk of bias or methodological quality for each included systematic review. | Pag. 9 |
|  | 18b | Present assessments (collected from systematic reviews or assessed anew) of the risk of bias of the primary studies included in the systematic reviews. | Not applicable |
|  | 18c | Present assessments of the risk of bias of supplemental primary studies (if included). | Not applicable |
| Summary or synthesis of results | 19a | For all outcomes, summarise the evidence from the systematic reviews and supplemental primary studies (if included). If meta-analyses were done, present for each the summary estimate and its precision and measures of statistical heterogeneity. If comparing groups, describe the direction of the effect. | Pag. 11-12-13 |
|  | 19b | If meta-analyses were done, present results of all investigations of possible causes of heterogeneity. | Not applicable |
|  | 19c | If meta-analyses were done, present results of all sensitivity analyses conducted to assess the robustness of synthesised results. | Not applicable |
| Reporting biases | 20 | Present assessments (collected from systematic reviews and/or assessed anew) of the risk of bias due to missing primary studies, analyses, or results in a summary or synthesis (arising from reporting biases at the levels of the systematic reviews, primary studies, and supplemental primary studies, if included) for each summary or synthesis assessed. | Not applicable |
| Certainty of evidence | 21 | Present assessments (collected or assessed anew) of certainty (or confidence) in the body of evidence for each outcome. | Pag. 12-13 |
| Discussion | | |  |
| Discussion | 22a | Summarise the main findings, including any discrepancies in findings across the included systematic reviews and supplemental primary studies (if included). | Pag. 13-14 |
|  | 22b | Provide a general interpretation of the results in the context of other evidence. | Pag. 13-14-15 |
|  | 22c | Discuss any limitations of the evidence from systematic reviews, their primary studies, and supplemental primary studies (if included) included in the overview of reviews. Discuss any limitations of the overview of reviews methods used. | Pag. 15 |
|  | 22d | Discuss implications for practice, policy, and future research (both systematic reviews and primary research). Consider the relevance of the findings to the end users of the overview of reviews, eg, healthcare providers, policymakers, patients, among others. | Pag. 14-15 |
| Other information | | |  |
| Registration and protocol | 23a | Provide registration information for the overview of reviews, including register name and registration number, or state that the overview of reviews was not registered. | Pag. 4 |
|  | 23b | Indicate where the overview of reviews protocol can be accessed, or state that a protocol was not prepared. | Pag. 4 |
|  | 23c | Describe and explain any amendments to information provided at registration or in the protocol. Indicate the stage of the overview of reviews at which amendments were made. | Supplementary File 2 |
| Support | 24 | Describe sources of financial or non-financial support for the overview of reviews, and the role of the funders or sponsors in the overview of reviews. | Pag. 18 |
| Competing interests | 25 | Declare any competing interests of the overview of reviews' authors. | Pag. 18 |
| Author information | 26a | Provide contact information for the corresponding author. | Pag. 1 |
|  | 26b | Describe the contributions of individual authors and identify the guarantor of the overview of reviews. | Pag. 18 |
| Availability of data and other materials | 27 | Report which of the following are available, where they can be found, and under which conditions they may be accessed: template data collection forms; data collected from included systematic reviews and supplemental primary studies; analytic code; any other materials used in the overview of reviews. | Pag. 18 |

# Supplementary File 2. EMethods

## Study design and methods

We conducted an umbrella review of SRs in accordance to the Cochrane Handbook’s chapter on overviews of reviews and the Joanna Briggs Institute Manual for Evidence Synthesis [1, 2]. We followed the Preferred Reporting Items for Systematic Reviews and Meta-Analyses (PRISMA) [3] for the flow chart and the Preferred Reporting Items for Overviews of Reviews (PRIOR) [4, 5]. The review protocol was registered in the International Prospective Register of Systematic Reviews (PROSPERO) database (CRD42022339140). Methods are summarized in Figure S1.


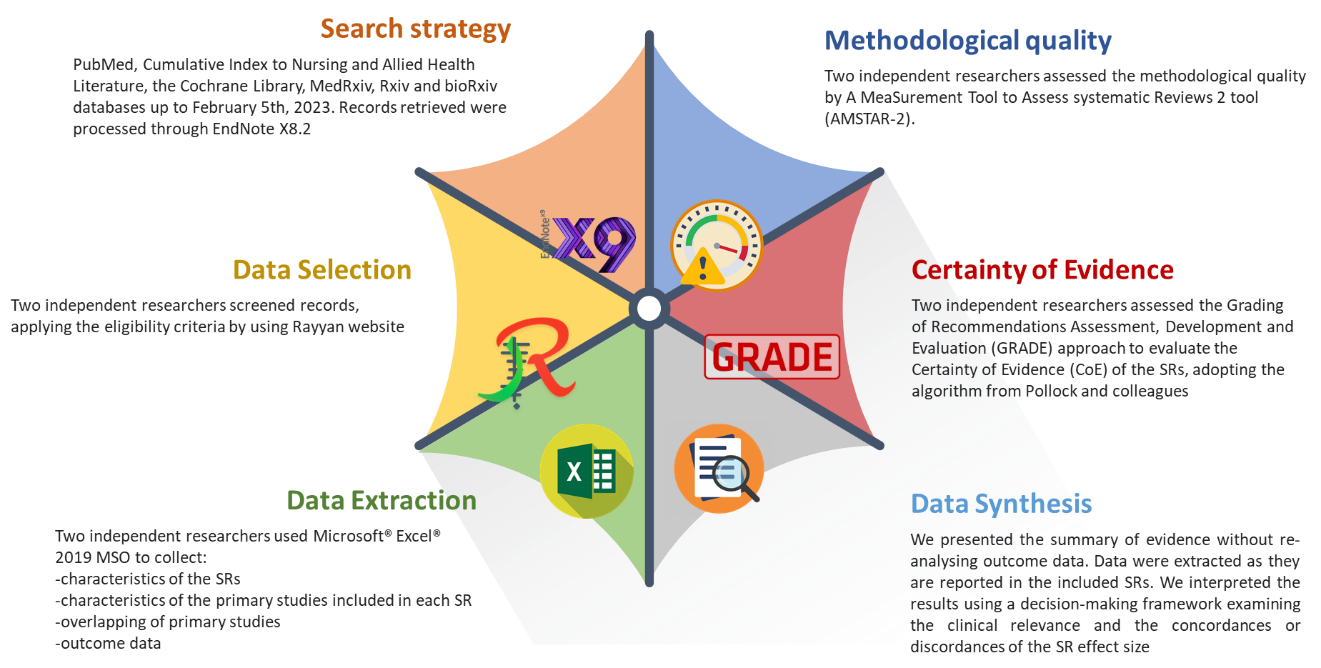


## Figure S1. Methods

## Patient and public involvement

Patients were not involved in this study.

## Equity, diversity, and inclusion statement

In this umbrella review the units of analysis are SRs. We assured equity, diversity and inclusion of the population as set in our eligibility criteria. Accordingly, we considered equity in the data collection, data analysis and interpretation of results. The author’s team is balanced in terms of gender and researchers (senior and junior).

## Difference between protocol and publication

In PROSPERO registration (CRD42022339140) we declared to search *PubMed, DARE (Database of Abstracts of Reviews of Effects), CINAHL (Cumulative Index to Nursing and Allied Health Literature), Cochrane Database of Systematic Reviews* but since the *DARE database* is already included in the Cochrane Library we run the search strategy in the following database: PubMed, CINAHL (Cumulative Index to Nursing and Allied Health Literature), and Cochrane Database of Systematic Reviews.

## Search strategy

In order to include the most updated evidence we investigated the following databases: PubMed, Cumulative Index to Nursing and Allied Health Literature, Cochrane Database of Systematic Reviews including the Database of Abstracts of Reviews of Effects (DARE). Both free and MeSH (Medical Subject Headings) terms were used. In addition, a free search through scientific websites (MedRxiv, Rxiv and bioRxiv databases) and the reference lists of retrieved articles was also performed.

***Databases***

*PubMed*

#1 "physical activit*"[All Fields]

#2 "exercis*"[All Fields]

#3 "exercise"[MeSH Terms]

#4 “sedentary behaviour]”[All Fields]

#5 “Sedentary Behavior"[MeSH Terms]

#6 "Obesity"[All Fields]

#7 Obesity"[MeSH Terms]

#8 #1 OR #2 OR #3 OR #4 OR #5 OR #6 OR #7

#9 "wearable device"[All Fields]

#10 "wearable electronic devices"[MeSH Terms]

#11 "wearable technology"[All Fields]

#12 "wrist-worn"[All Fields]

#13 "smartwatch"[All Fields]

#14 "wearable activity tracker"[All Fields]

#15 #9 OR #10 OR #11 OR #12 OR #13 OR #14

#16 "systematic reviews as topic"[MeSH Terms]

#17 "systematic review"[All Fields]

#18 "metanalysis"[All Fields]

#19 "meta analysis as topic"[MeSH Terms]

#20 "meta analysis"[All Fields]

#21 #16 OR #17 OR #18 OR #19 OR #20

#22 #8 AND #15 AND #21

*CINHAL*

| S1 | physical activit* |
| --- | --- |
| S2 | exercis* |
| S3 | (MH "Exercise+") |
| S4 | S1 OR S2 OR S3 |
| S5 | wearable device |
| S6 | wearable technology |
| S7 | wrist-worn |
| S8 | Smartwatch |
| S9 | wearable activity tracker |
| S10 | S5 OR S6 OR S7 OR S8 OR S9 |
| S11 | systematic review |
| S12 | Metanalysis |
| S13 | meta-analysis |
| S14 | (MH "Systematic Review") |
| S15 | (MH "Meta Analysis") |
| S16 | S11 OR S12 OR S13 OR S14 OR S15 |
| S17 | S4 AND S10 AND S16 |

*Cochrane*

#1 physical activit*

#2 exercis*

#3 MeSH descriptor: [Exercise] explode all trees

#4 sedentary behaviour*

#5 MeSH descriptor: [Sedentary Behavior] explode all trees

#6 obesity

#7 MeSH descriptor: [Obesity] explode all trees

#8 #1 OR #2 OR #3 OR #4 OR #5 OR #6 OR #7

#9 wearable device

#10 MeSH descriptor: [Wearable Electronic Devices] explode all trees

#11 wearable technology

#12 wrist-worn

#13 smartwatch

#14 wearable activity tracker

#15 #9 OR #10 OR #11 OR #12 OR #13 OR #14

#16 MeSH descriptor: [Systematic Reviews as Topic] explode all trees

#17 systematic review

#18 metanalysis

#19 meta analysis

#20 MeSH descriptor: [Meta-Analysis as Topic] explode all trees

#21 #16 OR #17 OR #18 OR #19 OR #20

#22 #8 AND #15 AND #21

***Repositories***

*medRxiv*

#1 physical activity

#2 exercise

#3 sedentary behaviour

#4 obesity

#5 wearable device

#6 wearable activity tracker

#7 wearable technology

#8 wrist-worn

#9 smartwatch

#10 systematic review

#11 metanalysis

#12 #1 AND #5 AND (#10 OR #11)

#13 #1 AND #6 AND (#10 OR #11)

#14 #1 AND #7 AND (#10 OR #11)

#15 #1 AND #8 AND (#10 OR #11)

#16 #1 AND #9 AND (#10 OR #11)

#17 #2 AND #5 AND (#10 OR #11)

#18 #2 AND #6 AND (#10 OR #11)

#19 #2 AND #7 AND (#10 OR #11)

#20 #2 AND #8 AND (#10 OR #11)

#21 #2 AND #9 AND (#10 OR #11)

#22 #3 AND #5 AND (#10 OR #11)

#23 #3 AND #6 AND (#10 OR #11)

#24 #3 AND #7 AND (#10 OR #11)

#25 #3 AND #8 AND (#10 OR #11)

#26 #3 AND #9 AND (#10 OR #11)

#27 #4 AND #5 AND (#10 OR #11)

#28 #4 AND #6 AND (#10 OR #11)

#29 #4 AND #7 AND (#10 OR #11)

#30 #4 AND #8 AND (#10 OR #11)

#31 #4 AND #9 AND (#10 OR #11)

*bioRxiv*

#1 physical activity

#2 exercise

#3 sedentary behaviour

#4 obesity

#5 wearable device

#6 wearable activity tracker

#7 wearable technology

#8 wrist-worn

#9 smartwatch

#10 systematic review

#11 metanalysis

#12 #1 AND #5 AND (#10 OR #11)

#13 #1 AND #6 AND (#10 OR #11)

#14 #1 AND #7 AND (#10 OR #11)

#15 #1 AND #8 AND (#10 OR #11)

#16 #1 AND #9 AND (#10 OR #11)

#17 #2 AND #5 AND (#10 OR #11)

#18 #2 AND #6 AND (#10 OR #11)

#19 #2 AND #7 AND (#10 OR #11)

#20 #2 AND #8 AND (#10 OR #11)

#21 #2 AND #9 AND (#10 OR #11)

#22 #3 AND #5 AND (#10 OR #11)

#23 #3 AND #6 AND (#10 OR #11)

#24 #3 AND #7 AND (#10 OR #11)

#25 #3 AND #8 AND (#10 OR #11)

#26 #3 AND #9 AND (#10 OR #11)

#27 #4 AND #5 AND (#10 OR #11)

#28 #4 AND #6 AND (#10 OR #11)

#29 #4 AND #7 AND (#10 OR #11)

#30 #4 AND #8 AND (#10 OR #11)

#31 #4 AND #9 AND (#10 OR #11)

*arXiv*

#1 physical activity

#2 exercise

#3 sedentary behaviour

#4 obesity

#5 wearable device

#6 wearable electronic devices

#7 wrist-worn

#8 smartwatch

#9 wearable activity tracker

#10 systematic review

#11 metanalysis

#14 #1 AND #10

#15 #2 AND #10

#16 #3 AND #10

#17 #4 AND #10

#18 #5 AND #10

#19 #6 AND #10

#20 #7 AND #10

#21 #8 AND #10

#22 #9 AND #10

#23 #1 AND #11

#24 #2 AND #11

#25 #3 AND #11

#26 #4 AND #11

#27 #5 AND #11

#28 #6 AND #11

#29 #7 AND #11

#30 #8 AND #11

#31 #9 AND #11

# Supplementary File 3. Methodological Quality and Certainty of the evidence

## Methodological Quality and Certainty of Evidence criteria

We appraised the methodological quality of each SR using the Assessment of Multiple Systematic Reviews, version 2 (AMSTAR 2), which rated quality as critically low, low, moderate, or high [6]. This tool allows for a reproducible critical evaluation of SRs of RCTs and NRSI in terms of an overall assessment of the reliability of the results included in the SRs.

The AMSTAR has 16 items in total. The overall rating is based on weaknesses in the following critical domain[6]:

- Protocol registered before commencement of the review (item 2)
- Adequacy of the literature search (item 4)
- Justification for excluding individual studies (item 7)
- Risk of bias from individual studies being included in the review (item 9)
- Appropriateness of meta-analytical methods (item 11)
- Consideration of risk of bias when interpreting the results of the review (item 13)
- Assessment of presence and likely impact of publication bias (item 15)

The overall confidence in the results of the review is rated as follows:

High - Zero or one non-critical weakness: The systematic review provides an accurate and comprehensive summary of the results of the available studies that address the question of interest

Moderate - More than one non-critical weakness*: The systematic review has more than one weakness, but no critical flaws. It may provide an accurate summary of the results of the available studies that were included in the review.

Low - One critical flaw with or without non-critical weaknesses: The review has a critical flaw and may not provide an accurate and comprehensive summary of the available studies that address the question of interest.

Critically low - More than one critical flaw with or without non-critical weaknesses: The review has more than one critical flaw and should not be relied on to provide an accurate and comprehensive summary of the available studies.

*Note: Multiple non-critical weaknesses may diminish confidence in the review and it may be appropriate to move the overall appraisal down from moderate to low confidence

We used the GRADE approach to evaluate the Certainty of Evidence (CoE) of the reviews for all outcomes in each treatment comparison adopting the algorithm developed for Cochrane overviews of reviews evaluating five dimensions (risk of bias, consistency of effect, imprecision, indirectness, and publication bias). In this algorithm, each review starts with a ranking of high certainty and is downgraded by 1 level for serious methodological concerns (sample size between 100 and 199 participants; high risk of bias in randomization and blinding for > 75% of included studies; high heterogeneity (I^2^> 75%); and “No” on one of these AMSTAR 2 items: a priori research design, comprehensive literature search, duplicate study selection, or duplicate data extraction) or 2 levels for very serious concerns (sample size < 100 participants and “No” on two or more of these AMSTAR2 items: a priori research design, comprehensive literature search, duplicate study selection, or duplicate data extraction) [7].

# Supplementary File 4. Clinical relevance

Clinical relevance was determined based on the guidelines proposed by Man-Son-Hing et al. (38), which considers the relationship between the minimal clinically important difference (MCID) of the treatment effect and the CI and designated to one of the following four different levels: (1) Definite – the MCID is smaller than the lower limit of the CI of the treatment effect, (2) Probable – the MCID is greater than the lower limit of the CI of the treatment effect, but smaller than the treatment effect, (3) Possible – the MCID is less than the upper limit of the CI of the treatment effect, but greater than the treatment effect, and (4) Definitely Not – the MCID is greater than the upper limit of the CI of the treatment effect. The CI should be based on the α value specified in the sample size calculation (Figure S1).

Chronological order of SR publication year was plotted, including the effect size of all meta-analyses in mean differences, to give an overall interpretation of steps per day, MVPA and SB. When effect sizes were reported in SMD, we first searched if back translations were already reported by SRs; otherwise, we back-translated them using the standard deviation of the control of the RCT with the highest number of participants of each meta-analysis [8].

## Figure S1. Framework for interpretation of clinical relevance

# Supplementary File 5. Excluded systematic reviews according to eligibility criteria

| Reference | Reason | Detail |
| --- | --- | --- |
| Albino de Queiroz D, André da Costa C, Aparecida Isquierdo Fonseca de Queiroz E, Folchini da Silveira E, da Rosa Righi R. Internet of Things in active cancer Treatment: A systematic review. J Biomed Inform. 2021 Jun;118:103814. doi: 10.1016/j.jbi.2021.103814. | No outcome of interest | Outcome: adverse effects and symptoms management, quality of life, survival |
| Cheatham SW, Stull KR, Fantigrassi M, Motel I. The efficacy of wearable activity tracking technology as part of a weight loss program: a systematic review. J Sports Med Phys Fitness. 2018 Apr;58(4):534-548. doi: 10.23736/S0022-4707.17.07437-0 | No outcome of interest | Outcome: weight loss |
| Coll F, Cavalheri V, Gucciardi DF, Wulff S, Hill K. Quantifying the Effect of Monitor Wear Time and Monitor Type on the Estimate of Sedentary Time in People with COPD: Systematic Review and Meta-Analysis. J Clin Med. 2022 Apr 1;11(7):1980. doi: 10.3390/jcm11071980. | No intervention of interest | Intervention: Monitor type and monitor wear time as moderators on the outcomes |
| Fraser MJ, Gorely T, O'Malley C, Muggeridge DJ, Giggins OM, Crabtree DR. Does Connected Health Technology Improve Health-Related Outcomes in Rural Cardiac Populations? Systematic Review Narrative Synthesis. Int J Environ Res Public Health. 2022 Feb 17;19(4):2302. doi: 10.3390/ijerph19042302. | No outcome of interest | Outcome: performance in an exercise capacity |
| Keogh A, Argent R, Anderson A, Caulfield B, Johnston W. Assessing the usability of wearable devices to measure gait and physical activity in chronic conditions: a systematic review. J Neuroeng Rehabil. 2021 Sep 15;18(1):138. doi: 10.1186/s12984-021-00931-2. | No outcome of interest | Outcome: usability of wearable devices |
| Kristoffersson A, Lindén M. A Systematic Review of Wearable Sensors for Monitoring Physical Activity. Sensors (Basel). 2022 Jan 12;22(2):573. doi: 10.3390/s22020573. | No design of interest | Not a review of efficacy of an intervention |
| Nuss K, Moore K, Nelson T, Li K. Effects of Motivational Interviewing and Wearable Fitness Trackers on Motivation and Physical Activity: A Systematic Review. Am J Health Promot. 2021 Feb;35(2):226-235. doi: 10.1177/0890117120939030. | No population of interest | Include subjects < 18 years |
| Ocagli H, Agarinis R, Azzolina D, Zabotti A, Treppo E, Francavilla A, Bartolotta P, Todino F, Binutti M, Gregori D, Quartuccio L. Physical activity assessment with wearable devices in rheumatic diseases: a systematic review and meta-analysis. Rheumatology (Oxford). 2022 Aug 25:keac476. doi: 10.1093/rheumatology/keac476. Online ahead of print. | No intervention of interest | Intervention: Monitor device types were compared (wrong control group) |
| Powell L, Parker J, Martyn St-James M, Mawson S. The Effectiveness of Lower-Limb Wearable Technology for Improving Activity and Participation in Adult Stroke Survivors: A Systematic Review. J Med Internet Res. 2016 Oct 7;18(10):e259. doi: 10.2196/jmir.5891. | No outcome of interest | Outcome: function of the lower limb |
| Prince SA, Cardilli L, Reed JL, Saunders TJ, Kite C, Douillette K, Fournier K, Buckley JP. A comparison of self-reported and device measured sedentary behaviour in adults: a systematic review and meta-analysis. Int J Behav Nutr Phys Act. 2020 Mar 4;17(1):31. doi: 10.1186/s12966-020-00938-3 | No intervention of interest | Intervention: Self-reported and devices were compared in estimating PA |
| Rodriguez-León C, Villalonga C, Munoz-Torres M, Ruiz JR, Banos O. Mobile and Wearable Technology for the Monitoring of Diabetes-Related Parameters: Systematic Review. JMIR Mhealth Uhealth. 2021 Jun 3;9(6):e25138. doi: 10.2196/25138. | No outcome of interest | Outcome: monitoring (not as an efficacy measure) of the diabetes related parameters |
| Sica M, Tedesco S, Crowe C, Kenny L, Moore K, Timmons S, Barton J, O'Flynn B, Komaris DS. Continuous home monitoring of Parkinson's disease using inertial sensors: A systematic review. PLoS One. 2021 Feb 4;16(2):e0246528. doi: 10.1371/journal.pone.0246528. | No outcome of interest | Outcome: monitoring (not as an efficacy measure) of the Parkinson related parameters |
| Soulard J, Carlin T, Knitza J, Vuillerme N. Wearables for Measuring the Physical Activity and Sedentary Behavior of Patients With Axial Spondyloarthritis: Systematic Review. JMIR Mhealth Uhealth. 2022 Aug 22;10(8):e34734. doi: 10.2196/34734. | No outcome of interest | Outcome: the use of wearable devices to measure PA or sedentary behaviour |
| Thanigaimani S, Jin H, Silva MT, Golledge J. Network Meta-Analysis of Trials Testing If Home Exercise Programs Informed by Wearables Measuring Activity Improve Peripheral Artery Disease Related Walking Impairment. Sensors (Basel). 2022 Oct 21;22(20):8070. doi: 10.3390/s22208070. | No outcome of interest | Outcome: change in walking distance |
| Yen HY, Chiu HL. The effectiveness of wearable technologies as physical activity interventions in weight control: A systematic review and meta-analysis of randomized controlled trials. Obes Rev. 2019 Oct;20(10):1485-1493. doi: 10.1111/obr.12909. | No outcome of interest | Outcome: weight |
| Burge AT, Cox NS, Abramson MJ, Holland AE. Interventions for promoting physical activity in people with chronic obstructive pulmonary disease (COPD). Cochrane Database Syst Rev. 2020 Apr 16;4(4):CD012626. doi: 10.1002/14651858.CD012626.pub2. | No intervention of interest | Intervention: every type of intervention to improve PA |
| Chastin S, Gardiner PA, Harvey JA, Leask CF, Jerez-Roig J, Rosenberg D, Ashe MC, Helbostad JL, Skelton DA. Interventions for reducing sedentary behaviour in community-dwelling older adults. Cochrane Database Syst Rev. 2021 Jun 25;6(6):CD012784. doi: 10.1002/14651858.CD012784.pub2. | No intervention of interest | Wrong comparison (mixing interventions) |
| Reeder B, David A. Health at hand: A systematic review of smart watch uses for health and wellness. J Biomed Inform. 2016 Oct;63:269-276. doi: 10.1016/j.jbi.2016.09.001 | No outcome of interest | Outcome: smart watch uses |
| Wang E, Abrahamson K, Liu PJ, Ahmed A. Can Mobile Technology Improve Weight Loss in Overweight Adults? A Systematic Review. West J Nurs Res. 2020 Sep;42(9):747-759. doi: 10.1177/0193945919888224. | No outcome of interest | Outcome: weight loss |
| Davergne T, Pallot A, Dechartres A, Fautrel B, Gossec L. Use of Wearable Activity Trackers to Improve Physical Activity Behavior in Patients With Rheumatic and Musculoskeletal Diseases: A Systematic Review and Meta-Analysis. Arthritis Care Res (Hoboken). 2019 Jun;71(6):758-767. doi: 10.1002/acr.23752. | No population of interest | Include subjects < 18 years |
| Freak-Poli R, Cumpston M, Albarqouni L, Clemes SA, Peeters A. Workplace pedometer interventions for increasing physical activity. Cochrane Database Syst Rev. 2020 Jul 21;7(7):CD009209. doi: 10.1002/14651858.CD009209.pub3. | No population of interest | Include subjects < 18 years |
| Hodkinson A, Kontopantelis E, Zghebi SS, Grigoroglou C, McMillan B, Marwijk HV, Bower P, Tsimpida D, Emery CF, Burge MR, Esmiol H, Cupples ME, Tully MA, Dasgupta K, Daskalopoulou SS, Cooke AB, Fayehun AF, Houle J, Poirier P, Yates T, Henson J, Anderson DR, Grey EB, Panagioti M. Association Between Patient Factors and the Effectiveness of Wearable Trackers at Increasing the Number of Steps per Day Among Adults With Cardiometabolic Conditions: Meta-analysis of Individual Patient Data From Randomized Controlled Trials. J Med Internet Res. 2022 Aug 30;24(8):e36337. doi: 10.2196/36337. | No population of interest | Include subjects < 18 years |
| Kang M, Marshall SJ, Barreira TV, Lee JO. Effect of pedometer-based physical activity interventions: a meta-analysis. Res Q Exerc Sport. 2009 Sep;80(3):648-55. doi: 10.1080/02701367.2009.10599604. | No population of interest | Include subjects < 18 years |
| Saunders DH, Mead GE, Fitzsimons C, Kelly P, van Wijck F, Verschuren O, Backx K, English C. Interventions for reducing sedentary behaviour in people with stroke. Cochrane Database Syst Rev. 2021 Jun 29;6(6):CD012996. doi: 10.1002/14651858.CD012996.pub2. | No intervention of interest | Intervention: every type of intervention to improve PA |
| Bahadori S, Collard S, Williams JM, Swain I. A review of current use of commercial wearable technology and smartphone apps with application in monitoring individuals following total hip replacement surgery. J Med Eng Technol. 2020 Aug;44(6):324-333. doi: 10.1080/03091902.2020.1797197. | No outcome of interest | Outcome: rehabilitation and patient monitoring |
| Romeo A, Edney S, Plotnikoff R, Curtis R, Ryan J, Sanders I, Crozier A, Maher C. Can Smartphone Apps Increase Physical Activity? Systematic Review and Meta-Analysis. J Med Internet Res. 2019 Mar 19;21(3):e12053. doi: 10.2196/12053. | No intervention of interest | Intervention: smartphone apps |
| Xu L, Shi H, Shen M, Ni Y, Zhang X, Pang Y, Yu T, Lian X, Yu T, Yang X, Li F. The Effects of mHealth-Based Gamification Interventions on Participation in Physical Activity: Systematic Review. JMIR Mhealth Uhealth. 2022 Feb 3;10(2):e27794. doi: 10.2196/27794. | No population of interest | Include subjects < 18 years |
| Khoo S, Mohbin N, Ansari P, Al-Kitani M, Müller AM. mHealth Interventions to Address Physical Activity and Sedentary Behavior in Cancer Survivors: A Systematic Review. Int J Environ Res Public Health. 2021 May 28;18(11):5798. doi: 10.3390/ijerph18115798. | No population of interest | Include subjects < 18 years |
| Abedtash H, Holden RJ. Systematic review of the effectiveness of health-related behavioral interventions using portable activity sensing devices (PASDs). J Am Med Inform Assoc. 2017 Sep 1;24(5):1002-1013. doi: 10.1093/jamia/ocx006. | No population of interest | Include subjects < 18 years |
| Ringeval M, Wagner G, Denford J, Paré G, Kitsiou S. Fitbit-Based Interventions for Healthy Lifestyle Outcomes: Systematic Review and Meta-Analysis. J Med Internet Res. 2020 Oct 12;22(10):e23954. doi: 10.2196/23954 | No population of interest | Include subjects < 18 years |
| Müller AM, Alley S, Schoeppe S, Vandelanotte C. The effectiveness of e-& mHealth interventions to promote physical activity and healthy diets in developing countries: A systematic review. Int J Behav Nutr Phys Act. 2016 Oct 10;13(1):109. doi: 10.1186/s12966-016-0434-2. | No population of interest | Include subjects < 18 years |
| Li X, Chen Z, Yue Y, Zhou X, Gu S, Tao J, Guo H, Zhu M, Du Q. Effect of Wearable Sensor-Based Exercise on Musculoskeletal Disorders in Individuals With Neurodegenerative Diseases: A Systematic Review and Meta-Analysis. Front Aging Neurosci. 2022 Jul 26;14:934844. doi: 10.3389/fnagi.2022.934844. | No outcome of interest | Outcomes: balance, other outcomes |
| Li C, Chen X, Bi X. Wearable activity trackers for promoting physical activity: A systematic meta-analytic review. Int J Med Inform. 2021 Aug;152:104487. doi: 10.1016/j.ijmedinf.2021.104487. | No population of interest | Include subjects < 18 years |
| Hodkinson A, Kontopantelis E, Adeniji C, van Marwijk H, McMillan B, Bower P, Panagioti M. (2019). Accelerometer- and Pedometer-Based Physical Activity Interventions Among Adults With Cardiometabolic Conditions: A Systematic Review and Meta-analysis. Journal of American Medical Association Network Open; 2(10):e1912895. doi: 10.1001/jamanetworkopen.2019.12895. Retraction in: Journal of American Medical Association Network Open. (2020); 3(12):e2032700. PMID: 31596494; PMCID: PMC6802237 | Other | Retracted |

# Supplementary File 6. General characteristics

## Table S1. Characteristics of included systematic reviews

| Author, year | Meta-analysis | N° of studies included | Population | Intervention | Control | Outcome related to physical activity |
| --- | --- | --- | --- | --- | --- | --- |
| *Mixed population* | | | | | | |
| Braakhuis, 2019 | Yes | 14 | Adults (over 21 years) that were patients of the health system | Wearable devices to offer objective feedback on physical activity | Standard treatment, interventions without objective feedback | Daily steps, walking time per day, energy expenditure (kJ or kcal per day or week), accelerometer count per day, time in moderate intensity physical activity per week |
| Bravata, 2007 | Yes | 26 | Adults in outpatient setting | Step goal and pedometer plus physical activity advice or usual care | Blind pedometer, usual care, another intervention to promote physical activity | Steps per day |
| Brickwood, 2019 | Yes | 28 | Adults (over  18 years) | Consumer-based wearable activity trackers on physical activity participation and sedentary behaviour (either as a single intervention or as part of a multi-component intervention) | Usual lifestyle, waitlist, blinded tracker, standard pedometer, standard behavioural group-based interventions, telephone counselling, smartphone app, education materials | Steps per day, MVPA (minutes/day or minutes/week), sitting time (%, hours/day, minutes/day), energy expenditure (kcal/week), activity units |
| Buckingham, 2019 | No | 25 | Workplace participants | Standalone mHealth or multi-component interventions (e.g., facilitated with telephone counselling) associated with BCTs | No intervention, no ‘true’ control group (the participants received at least a partial mHealth intervention), control supplied with wearable activity monitors for data  Collection, interventions without activity monitor | Physical activity (daily steps, daily or weekly minutes or MET, minutes of total activity or MVPA, exercise frequency, proportion of participants meeting step or physical activity goals, weekly minutes of light, moderate and vigorous intensity activity, walking distance and duration) Sedentary behaviour (daily or weekly sedentary time, computer activity) |
| Chaudhry, 2020 | Yes | 70 | Healthy adults, with pre-existing conditions, at risk of disease or without risk factors that prevent them from engaging in physical exertion | Any type of pedometer or other type of user’s device (physical or electronic) for counting steps, including smartphone app and common wearable fitness devices | Standard treatment, waiting list, health advice with minimal active involvement but without the use of a mobile device for counting steps (e.g., information leaflets, organizational support, physical activity and diet counselling, blind control, active lifestyle at work) | Daily step counting (pedometers, wearable monitors, smartphone application) |
| Daryabeygi-Khotbehsara, 2022 | No | 11 | Adults | Digital behaviour change interventions (delivered with smartphones) in which psychological theories are integrated with dynamic theories and computational models (e.g., control systems engineering) | Non-personalized generic recommendations, static suggestions, steady step  goals | Physical activity (change in daily step, total time spent in MVPA, stepping time) and sedentary behaviour |
| Delva, 2021 | No | 28 | Adults with cardiometabolic risk factor | Mobile health interventions  through websites, text messages, smartphone apps, voice technology, and digital medication tracking system | Usual care and other modalities (not reported) | Physical activity |
| Gal, 2018 | Yes | 18 | Adults | Activity tracker as a major component, combined with other components (e.g., education, counselling, goal setting) | Usual care, waiting list, education, counselling, exercise program, blinded activity tracker | Time spent in (MV)PA (minutes per day, mean number of steps per day), daily step count |
| Gierisch, 2015 | Yes | 14 | Adults (over 18 years) | Use of wearable activity devices that provided objective feedback about activity to the consumer (alone or in combination with other interventions aimed at promoting physical activity) | Standard treatment, waiting list, interventions based on pedometers, other active interventions focused on improving physical activity | Steps per day, total activity, percentage of participants who achieved physical activity goals |
| Goode, 2017 | Yes | 14 | Adults (healthy, older, overweight, sedentary, with chronic medical illness) | Wearable activity monitor as a major component of the intervention (the central motivational enhancement strategy) or a minor component (an integrated part of a suite of other motivation enhancement interventions, e.g., structured exercise program, behavioural counselling, or disease self-management techniques) | Inactive (e.g., waitlist, usual care) or active (e.g., group weight loss, counselling) | Physical activity (e.g., steps/day, hours/day, minutes/week, metabolic values) |
| Hakala, 2017 | Yes | 23 | Adults aged 18-65 years with cardiovascular  diseases, T2DM, breast cancer,  inactive, obese, healthy | Technologies that enabled interactive communication (e.g., phone calls, internet software/web pages), one-way communication from caregiver to participant (text messages, internet software/web pages), self-monitoring devices (e.g., accelerometer or pedometer) which were also used in combination with communicative technology | Physical activity recommendations only or treatment comparable to experimental group, but without the use of technology | Total physical activity (MET-hours per day or min/week) total energy expenditure (kcal per day), steps per day, active  counts (per minute/day), total physical activity counts (per minute/day or week), moderate exercise (MET min/week), MVPA (min/week) |
| Laranjo, 2021 | Yes | 35 | Adults (18 - 65 years) without chronic diseases | App or activity tracker capable of providing automatic and continuous self-monitoring and feedback on physical activity parameters in real time | No intervention (standard treatment, waiting list, intervention without app or monitor) or active control (control intervention that included mobile application or activity tracker) | Steps per day, MVPA (minutes / week), physical activity days per week, total physical activity (minutes / week), metabolic parameters (MET) (week) |
| Larsen, 2019 | Yes | 21 | Over 65 years old able to walk independently or without supports | Pedometer with the addition of other interventions (e.g., telephone consultations, education, calendar for self-monitoring of steps, definition of objectives) | Maintaining usual physical activity, group advice, standard treatment, other training, goal setting | Number of steps per day, number of meters per day, amount of energy expenditure per day (calories), MET per day (minutes or hours), self-reported physical activity (if objective data were not present); time spent as sedentary, time spent in MVPA) |
| Larsen, 2022 | Yes | 121 | 18- 65 years old | PAM with feedback | Intervention without feedback | Variations of physical activity, MVPA, sedentary time (step count, meters walked daily, daily energy expenditure) |
| Lewis, 2015 | No | 11 | Adults | Wearable device and other interventions (e.g., education, messages to provide feedback or motivation, telephone advice, paper diaries, brochures, web portal) | Normal physical activity behaviour, electronic activity monitoring systems but blind to feedback, passive treatment (e.g., provide physical activity literature, standard behavioural interventions for weight loss) | Sedentary behaviour (hours per day), moderate (MET per min / week) light (MET per min / week) or vigorous (MET per min / week) activity, leisure time spent on physical activity (MET per min / week), total physical activity (MET per min / week), energy expenditure (kcal per week), walking (MET per min / week) and steps (steps per day) |
| Lynch, 2020 | Yes | 21 | Adults (aged 18 and over) | Intervention with fitness tracker (alone or combined other interventions e.g., organizational wellness program, individualized goals, printed material, nutritional intervention) | Control (e.g., normal physical activity, nutrition guidelines, follow up calls, blinded fitness tracker) or alternative intervention (e.g., organizational wellness program, printed material, goal setting guide, financial incentive, weekly emails) | Time spent walking, minutes per day / week of light, moderate, or vigorous, MVPA (MET min / week or MET min / day), step count (steps per day), accelerometer count, expenditure estimate energy (kcal / week or kcal / day) |
| Qiu, 2015 | Yes | 15 | Adults | Step counters with goal setting and/or other components to improve physical activity (e.g., diet and exercise program, motivational website, cognitive behavioural program) | Usual care, were asked  to maintain current lifestyle or received interventions that had  nothing to do with physical activity or sedentary behaviour | Sedentary time |
| Stephenson, 2017 | Yes | 17 | Healthy or overweight / obese adults | Intervention with computer / mobile / wearable technology | Standard treatment, no intervention, waitlist, alternative treatment conditions (e.g., health information) | Reduction of sedentary behaviour |
| Tang, 2020 | Yes | 12 | Healthy adults | Wearable devices to provide the user with objective feedback regarding physical activity (alone or in combination with other interventions to improve physical activity) | Health related information, waiting list, pedometer, use of wearable device blinded and / or without feedback, standard intervention, routine physical activity, use of alternative intervention monitor | Steps per day, moderate to vigorous intensity total activity, activity unit or energy expenditure on physical activity |
| *Overweight or obese population* | | | | | | |
| de Vries, 2016 | Yes | 14 | Adults with overweight or obesity  (mean baseline BMI ≥ 27.0 kg/m2 for Caucasians or ≥ 25.0 kg/m2  for Asians) | Behavioural physical activity  interventions (e.g. goal setting, action planning, problem solving, education) with an activity monitor | Waitlist, usual care, behavioural physical activity  interventions without an activity monitor | Steps per day, total MVPA minutes per time, total walking MET-minutes per week |
| Fawcett, 2020 | No | 5 | Obese (BMI of 30 kg / m^2^ or more) or overweight (BMI 25 kg / m^2^ and 29.99 kg / m^2^) adults | Interventions included digital wearable technologies used for track or manage weight. Studies that only included a mobile phone app were not included | Traditional behavioural approaches for weight loss, routine care, or another intervention. Studies without a control group were also included if they met the other inclusion criteria | Change in physical activity after using digital wearable technology for at least one year |
| Dehghan Ghahfarokhi, 2022 | Yes | 26 | Overweight and/or obese adults | Wearable or smartphone app as a single part of the program, as the main emphasis of the  program but supported by physical activity counselling or  integrated as an essential component of a broader physical  activity program (with e.g., individualised physical activity prescription, social support) | Usual lifestyle, wearable device without app, no intervention, waitlist, goal setting | Steps per day, MVPA |
| Sypes, 2019 | No | 22 | Overweight or obese adults | Electronic activity monitoring systems associated with behaviour change techniques (e.g., goal setting, reinforcement) | No use of electronic activity monitoring systems, low similarity to the intervention (e.g., written or verbal information on a correct lifestyle, no intervention), medium similarity to the intervention (intervention similar to the intervention group but without electronic and non-electronic activity monitoring with the same extension as the intervention group), high similarity (same intervention except electronic activity monitoring systems) | Moderate activity (MET-min / week), vigorous activity (MET-min / week or minutes /day), MVPA (minutes / week), steps per day, MET-minutes / week overall, activity units |
| Wong, 2022 | Yes | 30 | Adults aged between 18 and 64 years who were obese (body mass index ≥30 kg/m^2^) or overweight (body mass index = 23–29.9 kg/m^2^) | Wearable device combined with other interventions (e.g., diet and exercise advice, meeting and support, feedback) | Placebo, standard care, active (diet and exercise) or passive interventions (e.g., information leaflet, waitlist, standard print material, self-monitoring booklet, group session, online newsletter) | Steps per day, steps per day change and physical activity intensity change (min/day) |
| *Older adults* | | | | | | |
| Cooper, 2018 | Yes | 9 | Adults (over 65 years) | Wearable devices combined with exercise consultancy,  definition of physical activity goals | Active intervention (e.g., self-monitoring, self-pacing, coaching, counselling), physical activity without pedometer, relying on self-reported measures to determine activity levels, self-monitoring of exercises with pedometers, but without counselling on guided exercises | Minutes spent walking per day, steps per day, activity units |
| Liu, 2020 | Yes | 10 | Elderly (over 55) with a sedentary lifestyle | Interventions in which an accelerometer or pedometer has been used to objectively measure daily steps or minutes in MVPA (e.g., consultations, prescribing and planning an exercise regimen) | Passive (no treatment / waitlist / minimal treatment e.g., standard treatment, health information) or active (alternative treatment with or without wearable device) control | Time spent in MVPA (minutes per day) and daily step count |
| Oliveira, 2020 | Yes | 23 | Elderly (over 60 or with an average age of at least 60 years) both healthy and with specific pathologies | Intervention based on activity tracking and with the aim of increasing participation in physical activity (activity monitor as a single component intervention or the main focus, but supported by physical activity counselling or incorporated as an important part of a broader intervention on physical activity) | No interventions, standard treatment, waiting list, standard flyer explaining the importance of physical activity, exercise and diet flyer, physical activity advice, physical activity counselling, education + blind pedometer | Steps per day, mobility as a secondary outcome related to physical activity (walking speed, sit to stand, functional tests, scales) |
| Yerrakalva, 2019 | Yes | 6 | Community-dwelling older adults (> 55 years) | App as primary focus of the intervention or in combination  with educational classes and phone calls with health care  professionals | No-content comparator group (e.g., waitlist, no intervention), nontechnology comparator group (physical exercise manual and paper log, educational sessions  without physical activity component), technology non-app comparator group (e.g., pedometer and self-monitoring) | Physical activity (minutes/day, steps/day), physical fitness (m/s, fastest gait speed, meters, 6-min timed walk, maximal oxygen uptake) or sedentary time (% sedentary time/day, sitting  time/day) |
| *Population with pathologies* | | | | | | |
| Armstrong, 2019 | Yes | 17 | Individuals with COPD | Standalone pedometer physical activity promotion intervention or alongside pulmonary rehabilitation | Usual care, pulmonary rehabilitation alone | Steps per day |
| Ashur, 2021 | Yes | 19 | Adults participating in cardiac rehabilitation programs | Wearable activity trackers and other interventions (e.g., consultation, tactile stimulation (vibration) in the case of sedentary behaviour, self-monitoring with goals on the number of steps to reach weekly) | Cardiac rehabilitation program without physical activity trackers, usual care | Daily step count and aerobic capacity (VO_2_max) |
| Baskerville, 2017 | Yes | 12 | Adults with T2DM | Activity monitors nested within a multifaceted intervention, activity monitors alone with minimal additional intervention | Same intervention but with blinded devices, educational material, routine care, "enhanced usual care" (e.g., usual care with advices) | Free-living physical activity |
| Blount, 2021 | No | 14 | Breast cancer survivors | Wearable activity trackers combined with health education interventions | Health Education Intervention on Facebook, standard care, no intervention | Physiological outcomes: physical activity levels, sit to stand, body weight, oxygen exchange and body composition, BMI, physical fatigue, energy expenditure, perceived physical health, blood biomarkers  Cognitive and emotional outcomes were then investigated |
| Chan, 2021 | No | 9 | Adults (>18 years) with sympthomatic intermittent claudication | Supervised exercise therapy in conjunction with a home-based exercise prescription with wearable activity monitor | Supervised exercise therapy and usual care or alternative | Changes in daily walking activity (steps per day, maximum ambulatory cadence) |
| de Leeuwerk, 2022 | Yes | 21 | Adults during hospitalization or inpatient  rehabilitation, or three months after it | Usual care combined with other interventions (e.g., a personalized program with self-monitoring; activity tracker, remote monitoring and social support, self-monitoring and physical activity with a physiotherapist's feedback), speed feedback and discussion of results and feedback with physiotherapist, exercise training at home with telemonitoring support, pedometer and counselling, a home-based program with pedometer and exercise counselling | Usual care, usual care associated with wearable device but without feedback, usual care and CBT, feedback without discussion of results, exercise training | Physical activity and physical functioning (steps per day, time spent walking, % of preoperative steps count, non-therapy walking time, minutes spent in MVPA, daily upright time, active minutes/day, inactive time, time spent in sedentary activity, TUG, cardiorespiratory fitness test, muscle strength, SPPB, POMA) |
| Franssen, 2020 | Yes | 35 | Adults (over 18 years) with major chronic diseases (e.g., chronic respiratory diseases, T2DM, cardiovascular diseases) | Consumer-based wearable activity monitor-based behaviour change intervention (alone or multi-component, e.g., in combination with lifestyle data platforms or apps, coaching sessions) | Control intervention (e.g., maintaining the same physical activity level, encouraging to increase the daily physical activity level, general information brochure, educational sessions, blinded device) or usual care | Number of steps per day |
| Hannan, 2019 | Yes | 9 | Adults (>20 years) with cardiac diseases who completed phase 2 of cardiac rehabilitation | Self-management using wearable physical activity monitors and goal setting (e.g., phone calls, emails, text messages or a web-based interface), cardiac rehabilitation with pedometer | Brochure, face-to-face sessions  on physical activity and lifestyle factors, weekly facilitator support without feedback, paper diary to report daily physical activity, advice to stay active, standard cardiac rehabilitation, wore wearable device only in the last weeks of the study | Step count/day, 6-MWT, sedentary behaviour |
| Hodkinson, 2021 | Yes | 38 | Adults with a diagnosis of T2DM, obesity, overweight, or cardiovascular disease | Interventions that used wearable activity trackers | Standard treatment | Combination of wearing a wearable activity monitor and physical activity levels (mean difference in physical activity levels) |
| Kamei, 2022 | Yes | 11 | Adults with chronic diseases (COPD, diabetes, CD) | Wearable device combined with an additional type of education intervention (e.g., goal setting, text messaging, written information) | Wearable devices without an educational intervention, usual care without a wearable device | Walking steps per day, duration of physical exercise (minutes) |
| Kirk, 2019 | Yes | 35 | Adults diagnosed with chronic cardio metabolic disease | 20% multicomponent intervention (wearable device plus another intervention e.g., other technology, diet) | Waiting list, standard treatment, improved standard treatment (with the addition of the device), active comparator (e.g., group vs basic) | Steps per day, MVPA (minutes), energy expenditure (kcal), VO_2_max (mL / [min x kg]), total physical activity (minutes/day), other (6-minute walk test, light activity physics, frequency) |
| Lee, 2022 | No | 33 | People diagnosed with any type of dementia | Information and communication technology device | Usual care, people with normal cognition | Physical activity |
| Lynch, 2018 | Yes | 4 | Adults diagnosed with stroke who were in an hospital setting or who lived in a community | Activity monitor alone or combined with another intervention | No intervention, other interventions, different activity monitors compared to the intervention, same intervention of the intervention group but without activity monitors | Steps per day, time spent in MVPA at the end of surgery, 3 months post-surgery and 12 months post-surgery (minutes per day, percentage of hours spent standing), MVPA (MET:> 3) or points activity cut-off (> 1952 per minute), sedentary time (minutes per day, % of walking hours), time spent in light-intensity physical activity (minutes per day, % of walking hours), walking duration (minutes per day, % of walking hours) |
| Mansi, 2014 | No | 7 | Adults with musculoskeletal diseases (knee osteoarthritis, CLBP, fibromyalgia) | Behavioural strategies to improve physical activity (e.g., goal setting,  problem solving, self-efficacy, and social support) and pedometer | Behavioural strategies to improve physical activity but without pedometer, behavioural strategies and pedometer without feedback | Steps per day |
| Master, 2022 | No | 6 | Adults who underwent orthopaedic surgical procedures (e.g., arthroplasty or arthrodesis) to manage musculoskeletal disorders | Pedometer along with other interventions (e.g., 12-week record chart prior to surgery, phone calls, steps/day goal, health coaching and financial incentives, home-based exercise program) | Wearable technology but with no feedback on their steps, steps progression, or counselling on physical activity goals; other interventions (e.g., in-person rehabilitation, general information on recovery and rehabilitation, home-based exercise program) | Physical activity (steps per day, time spent in MVPA), physical function (time needed to complete the TUG, 6-MWT and 4-meter walk test), pain, psychological distress, and general health (EQ-5D, KOOS, WOMAC, and SF-36) |
| Pudkasam, 2021 | Yes | 16 | Adult female breast cancer survivors diagnosed with stage  0 to III tumours | Pedometer combined with counselling, pedometer combined with motivational interviewing, pedometer combined with printed material, pedometer combined with social media, motivational interviewing | Usual care of breast cancer,  standard physical activity recommendation, waiting list | Exercise or physical activity adherence: weekly intensity of physical activity (MET), weekly minutes of MVPA, daily steps, % physical activity adherence |
| Qiu, 2014 | Yes | 11 | Outpatients with T2DM | Step counter with co-interventions (e.g., walking/activity program, diary, goal setting, telephone support) | Usual care, enhanced usual care (e.g., with education materials) step counters used only for counting steps | Steps per day |
| Qiu, 2018 | Yes | 15 | Adults with COPD (according to GOLD criteria) | Interventions in which the pedometer was the fundamental component for promoting physical activity | Usual care, interventions without pedometer or pedometer used only to record the number of steps per day | Steps per day, walking time, exercise capacity (6-MWT) |
| Rintala, 2018 | Yes | 11 | People with MS | Increasing  or promoting physical activity either with social-cognitive theory (e.g., goal setting, stretching manuals) or with a motivational interview (e.g., tailored physical activity, exercises based on individual needs in the participants’ daily life, physical abilities, environment resources, and motivation) | No treatment (e.g., wait-list), usual care (e.g., general advice on exercise, physical activity, or general advice excluding physical activity) minimal treatment (similar home DVD program as in the experimental group), or hippotherapy without the use of technology relating to distance physical rehabilitation | Total leisure physical activity (MET/min/week), total energy expenditure (kcal/kg/week) |
| Robinson, 2021 | No | 17 | Adult patients undergoing elective surgery | Preoperative interventions  (implemented before the surgical procedure); postoperative interventions  (implemented after the surgical procedure); and preoperative and postoperative interventions (implemented before and continued after operation) | Not reported | Minutes of vigorous activity per week |
| Schaffer, 2019 | No | 12 | Adults with cancer or survivors of adult-onset cancer | In-person exercise training component (e.g., supervised walking groups, training sessions at exercise facilities, educational sessions about E-DATs, dietary counselling); self-directed training component (e.g., exercise recommendations conveyed via phone with written materials, smartphone app) | Usual care or non-exercise interventions (e.g., phone calls, brochure), active control (e.g., flexibility activities, exercise recommendation) | Activity level (weekly physical activity, step count, MVPA, brisk walking, global daily physical activity, MET-hour per week, strenuous and mild intensity activity) |
| Singh, 2022 | Yes | 35 | People of diagnosis of cancer at any stage of treatment | Wearable physical activity tracker or pedometer, combined with a specific  behaviour change theory or model, baseline physical activity counselling or instruction session, (by phone or email) no in-person, phone or email-based counselling or support | Pedometer-based physical activity intervention during or following  a rehabilitation program, usual care | Post intervention physical activity levels (low-intensity physical activity, moderate-intensity physical activity, vigorous-intensity physical activity, MVPA, total physical activity, and  daily steps) |
| Vaes, 2013 | Yes | 24 | Adults with T2DM or COPD | Counselling (e.g., education, encouragement to improve physical activity, behaviour change strategies) associated with activity monitor | Usual care, usual care combined with counselling, no intervention | Steps per day, time spent walking, activity counts, activity intensity, frequency, and/or energy expenditure, MET*min/week, days/week following exercise, exercise capacity (6-MWT) |

**Note**: %: percentage; kcal: kilocalorie; MVPA: Moderate to Vigorous Physical Activity; MET: metabolic equivalent; PAM: Physical Activity Monitor; BMI: Body Mass Index; T2DM: Type 2 Diabetes Mellitus; VO_2_max: maximum oxygen uptake; TUG: Timed Up and Go test; 6-MWT: 6-Minute Walk Test; EQ-5D: EuroQol-5; KOOS: Knee injury and Osteoarthritis Outcome Score; WOMAC: Western Ontario and McMaster Universities Osteoarthritis Index; SF-36: Short Form Health Survey; COPD: Chronic Obstructive Pulmonary Disease; GOLD criteria: Global Initiative for Chronic Obstructive Lung Disease; CBT: Cognitive Behavioural Treatment; SPPB: Short Physical Performance Battery; POMA: Performed Oriented Mobility Assessment; CIPN: Chemotherapy-Induced Peripheral Neuropathy; CD: Cardiac Disease; MS: Multiple Sclerosis; BCT: Behavioural Cognitive Technique; mHealth: mobile health

## Table S2. Type of outcomes assessed in SR with meta-analysis

|  | Physical activity | | | Sedentary behaviour |
| --- | --- | --- | --- | --- |
|  | **Steps per day** | **MVPA per week** | **Composite** | **Minutes per day** |
| Armstong, 2019 | x |  |  |  |
| Ashur, 2021 | x |  |  |  |
| Baskerville, 2017 |  |  | x |  |
| Blount, 2021 |  |  |  |  |
| Braakhuis, 2019 |  |  |  |  |
| Bravata, 2007 | x |  |  |  |
| Brickwood, 2019 | x | x |  | x |
| Buckingham, 2019 |  |  |  |  |
| Chan, 2021 |  |  |  |  |
| Chaudhry, 2020 | x |  |  |  |
| Cooper, 2018 |  |  | x |  |
| Daryabeygi-Khotbehsara 2021 |  |  |  |  |
| De Leeuwerk, 2022 |  |  | x |  |
| De Vries, 2016 | x | x |  |  |
| Dehghan Ghahfarokhi, 2022 | x | x |  |  |
| Delva, 2021 |  |  |  |  |
| Fawcett, 2020 |  |  |  |  |
| Frassen, 2020 | x |  |  |  |
| Gal, 2018 | x | x |  |  |
| Gierisch, 2015 |  |  | x |  |
| Goode, 2017 |  |  | x |  |
| Hakala, 2017 |  |  | x* |  |
| Hannan, 2019 | x |  |  |  |
| Hodkinson, 2021 |  |  | x |  |
| Kamei, 2022 | x |  |  |  |
| Kirk, 2019 | x | x |  |  |
| Laranjo, 2021 |  |  | x |  |
| Larsen, 2019 |  | x | x | x |
| Larsen, 2022 |  | x | x | x |
| Lee, 2022 |  |  |  |  |
| Lewis, 2015 |  |  |  |  |
| Liu, 2020 | x | x |  |  |
| Lynch, 2018 | x | x |  |  |
| Lynch, 2020 | x | x |  |  |
| Mansi, 2014 |  |  |  |  |
| Master, 2022 |  |  |  |  |
| Pudkasam, 2021 | x | x |  |  |
| Qiu, 2014 | x |  |  |  |
| Qiu, 2015 |  |  |  | x |
| Qiu, 2018 |  |  | x |  |
| Rintala, 2018 |  |  | x |  |
| Robinson, 2021 |  |  |  |  |
| S Oliveira, 2020 | x |  |  |  |
| Schaffer, 2019 |  |  |  |  |
| Singh, 2022 | x* | x* | x* |  |
| Stephenson, 2017 |  |  |  | x |
| Sypes, 2019 |  |  |  |  |
| Tang, 2020 | x |  | x |  |
| Vaes, 2013 |  |  | x |  |
| Wong, 2022 | x | x |  |  |
| Yerrakalva, 2019 | x |  |  | x |

Legend: *SRs were not considered due to different outcome measurements of effect sizes (RR) and missing

references; SB, Sedentary Behaviour; MVPA, Moderate to Vigorous Physical Activity.

^ composite measurements of physical activity (e.g., metabolic equivalent for task (MET), min/week, intensity, time spent walking)

## Table S3. Type of outcomes assessed in SR without meta-analysis

|  | Physical activity | | | Sedentary behaviour |
| --- | --- | --- | --- | --- |
|  | Steps per day | MVPA | Composite |  |
| Blount, 2021 | **x** | x |  |  |
| Buckingham, 2019 | x | x |  | x |
| Chan, 2021 | x |  |  |  |
| Daryabeygi-Khotbehsara 2021 | x | x |  | x |
| Delva, 2021 | x |  |  |  |
| Fawcett, 2020 | x | x |  |  |
| Lee, 2022 |  |  | x |  |
| Lewis, 2015 | x |  |  | x |
| Mansi, 2014 |  |  | x |  |
| Master, 2022 | x | x |  |  |
| Robinson, 2021 | x | x |  |  |
| Schaffer, 2019 | x |  |  |  |
| Sypes, 2019 | x | x |  |  |

SB, Sedentary Behaviour; MVPA, Moderate to Vigorous Physical Activity.

^ composite measurements of physical activity (e.g., metabolic equivalent for task (MET), min/week, intensity, time spent walking)

#

# Supplementary File 7. Methodological quality in systematic reviews in accordance with AMSTAR 2

The primary critical weaknesses corresponded to not providing a list of excluded studies with a justification of the reasons (n=44), not using a comprehensive literature search strategy (n=29), and not justifying the choice of meta-analysis as an appropriate tool for the statistical combination of results (n=19). The most frequent flaws of non-critical weaknesses were not reporting the sources of funding of the studies included in the SRs (n=46), not motivating the choice of the design of the studies included in the SRs (n=41), and not performing the extraction data by at least two independent authors (n=3). AMSTAR 2 assessments for each SR are reported in Table S1. In Figure S1 we reported the methodological quality of the 51 SR according to the 16 items of AMSTAR 2

# Table S1. AMSTAR 2 of each included SR

| Author, year | 1 | 2 | 3 | 4 | 5 | 6 | 7 | 8 | 9 | 10 | 11 | 12 | 13 | 14 | 15 | 16 | Confidence |
| --- | --- | --- | --- | --- | --- | --- | --- | --- | --- | --- | --- | --- | --- | --- | --- | --- | --- |
| Armstrong, 2019 | Y | Y | N | N | Y | Y | N | Y | Y | N | N | Y | Y | Y | N | Y | CL |
| Ashur, 2021 | Y | Y/N | N | N | Y | N | N | Y | Y | N | N | Y | Y | Y | Y | N | CL |
| Baskerville, 2017 | Y | Y/N | N | N | Y | Y | N | Y | Y/N | N | Y | N | N | Y | Y | Y | CL |
| Blount, 2021 | Y | N | N | Y/N | N | Y | N | Y/N | Y | N | NM | NM | Y | Y | NM | Y | CL |
| Braakhuis, 2019 | Y | N | N | N | Y | N | N | Y | Y | N | N | Y | Y | Y | N | Y | CL |
| Bravata, 2007 | Y | N | Y | N | N | Y | N | Y | N | N | N | N | N | N | Y | Y | CL |
| Brickwood, 2019 | Y | N | S | Y | Y | Y | N | Y/N | Y | N | N | Y | Y | Y | N | Y | CL |
| Buckingham, 2019 | Y | Y | Y | Y/N | Y | N | N | Y | Y | N | NM | NM | Y | Y | NM | Y | L |
| Chan, 2021 | N | N | N | Y/N | Y | N | N | Y | Y | N | NM | NM | N | Y | NM | Y | CL |
| Chaudhry, 2020 | Y | Y | N | Y/N | Y | Y | N | Y | Y | Y | Y | Y | Y | N | Y | Y | L |
| Cooper, 2018 | Y | N | N | N | Y | Y | N | Y/N | Y | N | N | Y | Y | Y | Y | Y | CL |
| Daryabeygi-Khotbehsara, 2021 | Y | Y | Y | N | Y | Y | Y | Y | Y | N | NM | NM | Y | Y | NM | Y | L |
| de Leeuwerk, 2022 | Y | Y | N | Y/N | Y | N | N | Y | Y | N | Y | Y | N | Y | Y | Y | CL |
| de Vries, 2016 | Y | Y | N | N | Y | Y | N | Y/N | Y | N | N | N | N | N | Y | N | CL |
| Dehghan Ghahfarokhi, 2022 | Y | N | Y | N | Y | Y | N | Y | Y | N | Y | Y | Y | Y | Y | Y | CL |
| Delva, 2021 | Y | N | Y | Y/N | Y | N | N | N | Y | N | NM | NM | Y | Y | NM | Y | CL |
| Fawcett, 2020 | Y | Y/N | N | Y/N | Y | N | Y | N | Y | N | NM | NM | Y | Y | NM | Y | M |
| Franssen, 2020 | Y | Y | N | N | Y | Y | N | Y | Y | N | N | Y | Y | Y | Y | Y | CL |
| Gal, 2018 | Y | Y | N | Y/N | Y | Y | N | Y | Y | N | Y | Y | Y | Y | Y | Y | L |
| Gierisch, 2015 | Y | Y/N | N | Y/N | Y | Y | N | Y/N | Y | Y | N | Y | Y | Y | Y | Y | CL |
| Goode, 2017 | Y | Y/N | N | Y/N | Y | Y | N | Y | Y | N | Y | Y | Y | Y | N | Y | CL |
| Hakala, 2017 | Y | Y | N | N | Y | N | N | Y | Y | N | N | N | N | Y | N | Y | CL |
| Hannan, 2019 | Y | Y/N | N | Y/N | Y | Y | N | Y | Y | N | Y | Y | Y | Y | N | Y | CL |
| Hodkinson, 2021 | Y | Y | Y | Y | N | N | N | N | Y | N | N | Y | N | N | Y | Y | CL |
| Kamei, 2022 | Y | N | N | N | Y | N | N | Y | Y | N | N | Y | Y | Y | N | Y | CL |
| Kirk, 2019 | Y | N | N | N | N | Y | N | Y/N | Y | N | N | Y | Y | Y | Y | Y | CL |
| Laranjo, 2021 | Y | Y | N | Y/N | Y | N | Y | Y | Y | Y | Y | Y | Y | Y | Y | Y | M |
| Larsen, 2019 | Y | Y | N | Y | Y | Y | Y | Y | Y | Y | N | Y | Y | Y | Y | Y | L |
| Larsen, 2022 | Y | Y | N | Y/N | Y | Y | N | Y | Y | N | Y | Y | Y | Y | Y | Y | L |
| Lee, 2022 | Y | Y/N | N | N | Y | N | N | N | N | N | NM | NM | N | N | NM | Y | CL |
| Lewis, 2015 | Y | N | N | N | Y | N | Y/N | Y | N | N | NM | NM | Y | Y | NM | Y | CL |
| Liu, 2020 | Y | Y/N | N | N | Y | N | N | Y | Y | N | N | Y | Y | Y | N | Y | CL |
| Lynch, 2018 | Y | Y | N | Y | Y | Y | Y | Y | Y | N | Y | Y | Y | Y | Y | Y | M |
| Lynch, 2020 | Y | N | N | N | N | Y | N | Y | Y | N | Y | Y | Y | Y | N | Y | CL |
| Mansi, 2014 | Y | N | Y | N | Y | Y | N | Y | Y/N | Y | NM | NM | Y | N | N | Y | CL |
| Master, 2022 | Y | Y | Y | N | Y | N | N | N | Y | N | NM | NM | Y | N | NM | Y | CL |
| Pudkasam, 2021 | Y | Y | N | Y/N | Y | Y | N | Y | Y | N | Y | Y | Y | Y | Y | Y | L |
| Qiu, 2014 | Y | Y | N | N | Y | N | N | Y | Y | N | N | Y | Y | Y | Y | Y | CL |
| Qiu, 2015 | Y | Y | N | N | Y | Y | N | Y | Y | N | N | Y | Y | Y | Y | Y | CL |
| Qiu, 2018 | Y | Y | N | N | Y | N | N | Y | Y | N | Y | Y | N | Y | Y | Y | CL |
| Rintala, 2018 | Y | Y | N | N | Y | N | N | Y | Y | N | N | N | Y | Y | Y | Y | CL |
| Robinson, 2021 | Y | Y/N | N | Y/N | Y | N | N | N | Y | N | NM | NM | Y | Y | NM | Y | L |
| S Oliveira, 2020 | Y | Y | N | Y/N | Y | Y | N | Y | Y | N | NM | Y | Y | Y | Y | Y | L |
| Schaffer, 2019 | Y | N | N | Y/N | Y | Y | N | Y | N | N | NM | NM | N | Y | NM | Y | CL |
| Singh, 2022 | Y | Y/N | N | N | N | N | N | Y/N | Y | N | N | N | Y | Y | N | Y | CL |
| Stephenson, 2017 | Y | Y/N | N | N | N | N | N | Y | Y | N | Y | Y | Y | Y | N | Y | CL |
| Sypes, 2019 | Y | N | N | N | Y | N | N | Y/N | Y | N | NM | NM | Y | Y | NM | Y | CL |
| Tang, 2020 | Y | Y | N | N | N | Y | N | Y | Y | N | Y | Y | Y | Y | Y | Y | CL |
| Vaes, 2013 | Y | N | N | N | Y | N | N | Y | Y | N | Y | N | N | Y | Y | Y | CL |
| Wong, 2022 | Y | Y | Y | N | Y | Y | Y | Y | Y | N | Y | Y | Y | Y | Y | Y | L |
| Yerrakalva, 2019 | Y | Y/N | N | Y/N | Y | Y | N | Y | Y | N | Y | Y | Y | Y | Y | Y | L |

**Legend**. Y: Yes; N: no; Y/N: Partially yes; NM: No meta-analysis; H: High confidence; M: Moderate confidence; L: Low confidence; CL: Critically low confidence; 1: item 1 (Did the research questions and inclusion criteria for the review include the components of PICO?); 2: item 2 (Did the report of the review contain an explicit statement that the review methods were established prior to the conduct of the review and did the report justify any significant deviations from the protocol?); 3: item 3 (Did the review authors explain their selection of the study designs for inclusion in the review?); 4: item 4 (Did the review authors use a comprehensive literature search strategy?) 5: item 5 (Did the review authors perform study selection in duplicate?); 6: item 6 (Did the review authors perform data extraction in duplicate?); 7: item 7 (Did the review authors provide a list of excluded studies and justify the exclusions?); 8: item 8 (Did the review authors describe the included studies in adequate detail?); 9: item 9 (Did the review authors use a satisfactory technique for assessing the risk of bias (RoB) in individual studies that were included in the review?); 10: item 10 (Did the review authors report on the sources of funding for the studies included in the review?); 11: item 11 (If meta-analysis was performed, did the review authors use appropriate methods for statistical combination of results?); 12: item 12 (If meta-analysis was performed, did the review authors assess the potential impact of RoB in individual studies on the results of the meta-analysis or other evidence synthesis?); 13: item 13 (Did the review authors account for RoB in primary studies when interpreting/discussing the results of the review?); 14: item 14 (Did the review authors provide a satisfactory explanation for, and discussion of, any heterogeneity observed in the results of the review?); 15: item 15 (If they performed quantitative synthesis did the review authors carry out an adequate investigation of publication bias (small study bias) and discuss its likely impact on the results of the review?); 16: item 16 (Did the review authors report any potential sources of conflict of interest, including any funding they received for conducting the review?)

**
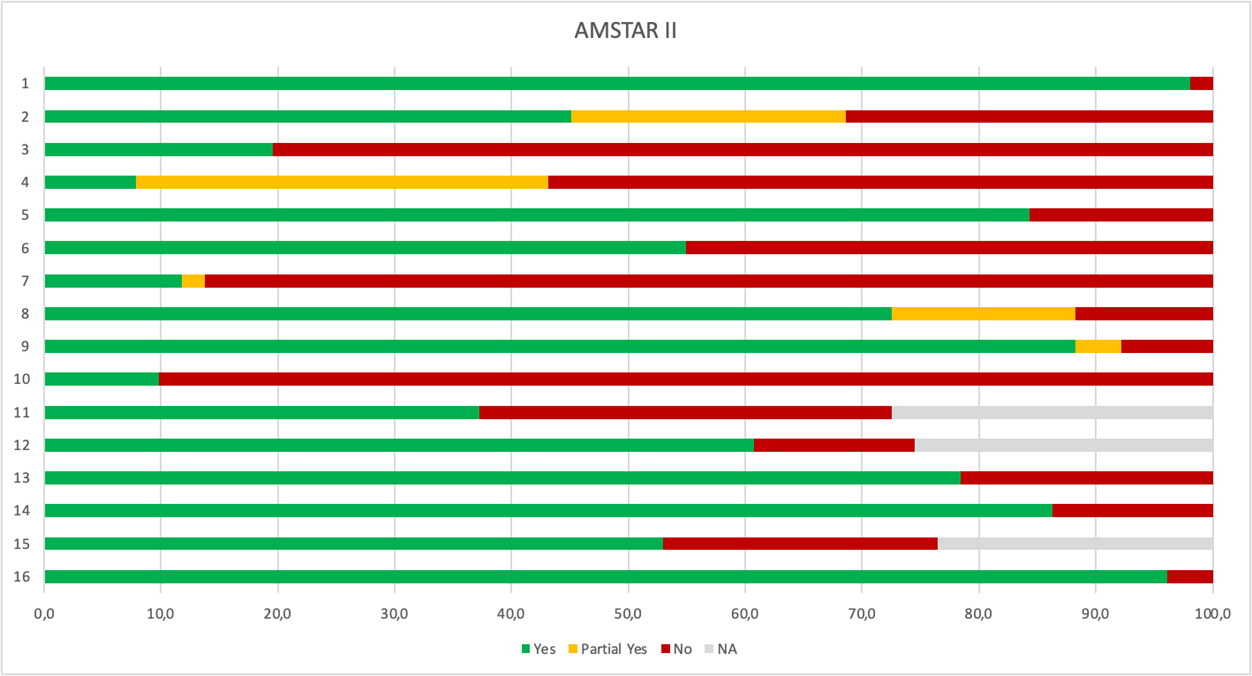
**

## Figure S1. Methodological quality of the 51 SR according to the 16 items of AMSTAR 2

**Legend.** 1**:**item 1 (Did the research questions and inclusion criteria for the review include the components of PICO?); 2: item 2 (Did the report of the review contain an explicit statement that the review methods were established prior to the conduct of the review and did the report justify any significant deviations from the protocol?); 3: item 3 (Did the review authors explain their selection of the study designs for inclusion in the review?); 4: item 4 (Did the review authors use a comprehensive literature search strategy?) 5: item 5 (Did the review authors perform study selection in duplicate?); 6: item 6 (Did the review authors perform data extraction in duplicate?); 7: item 7 (Did the review authors provide a list of excluded studies and justify the exclusions?); 8: item 8 (Did the review authors describe the included studies in adequate detail?); 9: item 9 (Did the review authors use a satisfactory technique for assessing the risk of bias (RoB) in individual studies that were included in the review?); 10: item 10 (Did the review authors report on the sources of funding for the studies included in the review?); 11: item 11 (If meta-analysis was performed, did the review authors use appropriate methods for statistical combination of results?); 12: item 12 (If meta-analysis was performed, did the review authors assess the potential impact of RoB in individual studies on the results of the meta-analysis or other evidence synthesis?); 13: item 13 (Did the review authors account for RoB in primary studies when interpreting/discussing the results of the review?); 14: item 14 (Did the review authors provide a satisfactory explanation for, and discussion of, any heterogeneity observed in the results of the review?); 15: item 15 (If they performed quantitative synthesis did the review authors carry out an adequate investigation of publication bias (small study bias) and discuss its likely impact on the results of the review?); 16: item 16 (Did the review authors report any potential sources of conflict of interest, including any funding they received for conducting the review?)

# Supplementary File 8. Systematic Review without Meta-Analysis

We found sparse effects that favoured WD from primary studies included in the 13 SRs without meta-analysis (Table S1). The most reported outcomes were PA (generically and inconsistently defined) and SB. On average, the proportion of trials reporting statistically significant results were of 56% (95%CI 0.23%-0.81%) and 32% (95%CI 0.11%-0.69%), respectively (Figure S1).

# Table S1. Findings in Systematic Review without Meta-Analysis

| Id | SR Without Ma | Outcome | Outcome Measurement | Sample Size | N. of Trials With Positive Findings Between Groups | Population |
| --- | --- | --- | --- | --- | --- | --- |
| 1 | Blount, 2021 | PA | Steps per day and MPVA | 11 | 8 | Population with pathologies |
| 2 | Buckingham, 2019 | SB | SB | 10 | 4 | Mixed population |
| 2 | Buckingham, 2019 | PA | Steps per day and MPVA | 25 | 14 | Mixed population |
| 3 | Chan, 2021 | PA | Steps per day | 4 | 1 | Population with pathologies |
| 4 | Daryabeygi-Khotbehsara 2021 | SB | SB | Not Reported | Not Reported | Mixed population |
| 4 | Daryabeygi-Khotbehsara 2021 | PA | Steps per day and MPVA | 6 | 5 | Mixed population |
| 5 | Delva, 2021 | PA | Steps per day | 6 | 4 | Mixed population |
| 6 | Fawcett, 2020 | PA | Steps per day and MPVA | 3 | 1 | Overweight or obese population |
| 7 | Lee, 2022 | PA | Steps per day and Other | Not Reported | Not Reported | Population with pathologies |
| 8 | Lewis, 2015 | PA | Steps per day | 9 | 1 | Mixed population |
| 8 | Lewis, 2015 | SB | SB | 4 | 1 | Mixed population |
| 9 | Mansi, 2014 | PA | Steps per day and Other | 7 | 4 | Population with pathologies |
| 10 | Master, 2022 | PA | MPVA | 2 | 0 | Population with pathologies |
| 10 | Master, 2022 | PA | Steps per day | 5 | 3 | Population with pathologies |
| 11 | Robinson, 2021 | PA | Steps per day and MPVA | Not Reported | Not Reported | Population with pathologies |
| 12 | Schaffer, 2019 | PA | Steps per day | 5 | 3 | Population with pathologies |
| 13 | Sypes, 2019 | PA | Steps per day and MPVA | 20 | 4 | Overweight or obese population |

**Legend**: PA, Physical Activity; MVPA, Moderate to Vigorous Physical Activity; SB, Sedentary Behavior; SR, Systematic Review

## Figure S1. Proportion of trials with positive results in SRs without meta-analysis

**Legend.** PA, physical activity; SB, Sedentary behaviour; SR, Systematic review
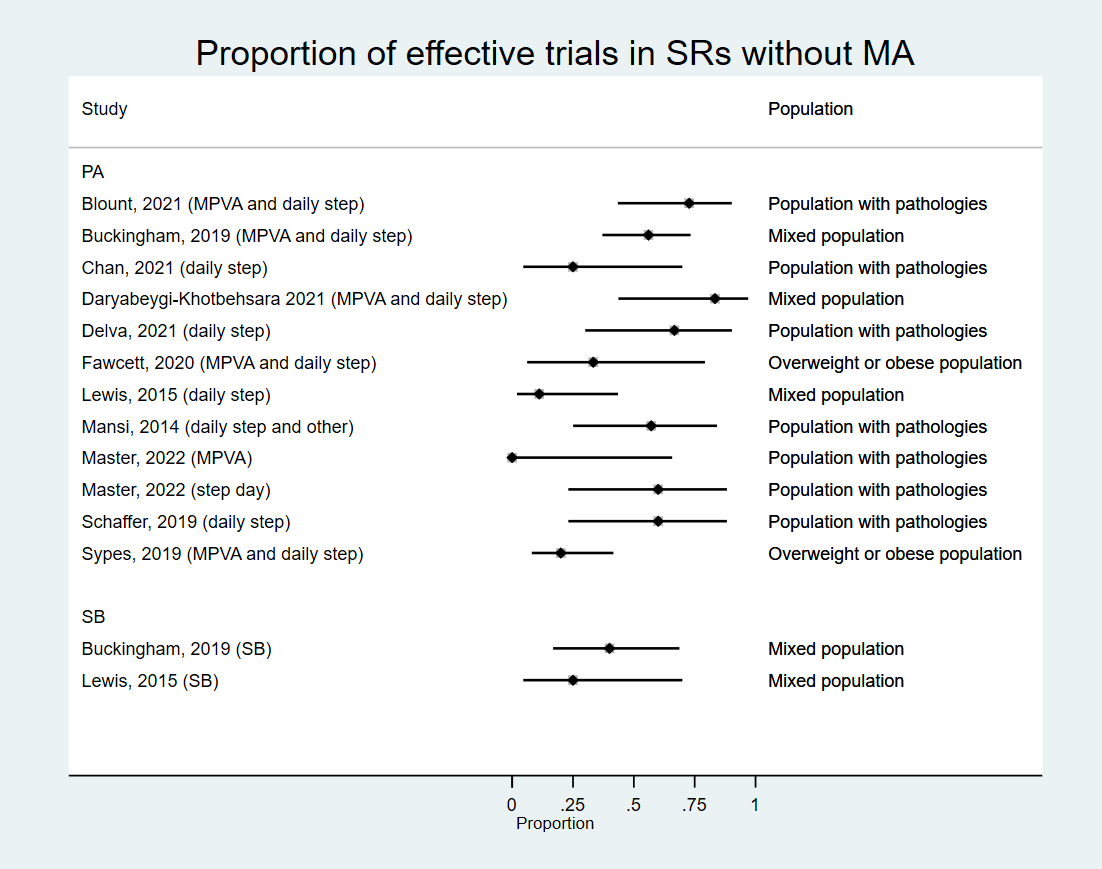


# Supplementary File 9. Overlapping Corrected Covered Area (CCA)

## Descriptive characteristics of overlapping

We did not consider two SRs from overlapping across outcomes due to different reasons. One SR performed one meta-analysis of continuous outcome with an unplanned measure of effect (Risk Ratio [RR]) (39). One SR performed three meta-analysis not providing the references of included primary studies (40). In Table S1 we reported the overlapping across outcomes by population. In Figure S1-S4 we reported CCA analysis for each outcome by subgroups.

**Table S1**. Subgroup of population of overlapping

| Population | Step day | MVPA | Composite measurements | Sedentary Behaviour |
| --- | --- | --- | --- | --- |
| Mixed population | Moderate overlap | Slight overlap | Slight overlap | Slight overlap |
| People with pathologies | Slight overlap | Slight overlap | Slight overlap | * |
| Older adults | Slight overlap | Slight overlap | One SR | One SR |
| Obese or overweight | Moderate overlap | Slight overlap | * | * |

**Legend**: MVPA, Moderate to Vigorous Physical Activity.

Note: *no SR assessed the outcome

## Figure S1. Physical activity as Steps per day

Steps per day. Mixed population

| Overall results | | |
| --- | --- | --- |
|  |  |  |
| Number of columns (number of reviews) | c | 6 |
| Number of rows (number of index publications) | r | 43 |
| Number of included primary studies (including double counting) | N | 58 |
| Covered area | N/(rc) | 22,48% |
| Corrected covered area | (N-r)/(rc-r) | 6,98% |
| Interpretation of overlap | Moderate overlap | |
| Structural Zeros | X | 0 |
| Corrected covered area  (adjusting by structural zeros) | (N-r)/(rc-r-X) | 6,98% |

Steps per day. People with pathologies

| Overall results | | |
| --- | --- | --- |
|  |  |  |
| Number of columns (number of reviews) | c | 9 |
| Number of rows (number of index publications) | r | 64 |
| Number of included primary studies (including double counting) | N | 80 |
| Covered area | N/(rc) | 13,89% |
| Corrected covered area | (N-r)/(rc-r) | 3,13% |
| Interpretation of overlap | Slight overlap | |
| Structural Zeros | X | 0 |
| Corrected covered area  (adjusting by structural zeros) | (N-r)/(rc-r-X) | 3,13% |

Steps per day. Older adults

| Overall results | | |
| --- | --- | --- |
|  |  |  |
| Number of columns (number of reviews) | c | 3 |
| Number of rows (number of index publications) | r | 26 |
| Number of included primary studies (including double counting) | N | 28 |
| Covered area | N/(rc) | 35,90% |
| Corrected covered area | (N-r)/(rc-r) | 3,85% |
| Interpretation of overlap | Slight overlap | |
| Structural Zeros | X | 0 |
| Corrected covered area  (adjusting by structural zeros) | (N-r)/(rc-r-X) | 3,85% |

Steps per day. Obese or overweight people

| Overall results | | |
| --- | --- | --- |
|  |  |  |
| Number of columns (number of reviews) | c | 3 |
| Number of rows (number of index publications) | r | 26 |
| Number of included primary studies (including double counting) | N | 31 |
| Covered area | N/(rc) | 39,74% |
| Corrected covered area | (N-r)/(rc-r) | 9,62% |
| Interpretation of overlap | Moderate overlap | |
| Structural Zeros | X | 0 |
| Corrected covered area  (adjusting by structural zeros) | (N-r)/(rc-r-X) | 9,62% |

## Figure S2. Physical activity as moderate to Vigorous Physical Activity (MVPA)

MVPA. Mixed population

| Overall results | | |
| --- | --- | --- |
|  |  |  |
| Number of columns (number of reviews) | c | 5 |
| Number of rows (number of index publications) | r | 79 |
| Number of included primary studies (including double counting) | N | 90 |
| Covered area | N/(rc) | 22,78% |
| Corrected covered area | (N-r)/(rc-r) | 3,48% |
| Interpretation of overlap | Slight overlap | |
| Structural Zeros | X | 0 |
| Corrected covered area  (adjusting by structural zeros) | (N-r)/(rc-r-X) | 3,48% |

MVPA. People with pathologies

| **Overall results** | | |
| --- | --- | --- |
|  |  |  |
| Number of columns (number of reviews) | c | 3 |
| Number of rows (number of index publications) | r | 23 |
| Number of included primary studies (including double counting) | N | 23 |
| Covered area | N/(rc) | 33,33% |
| Corrected covered area | (N-r)/(rc-r) | 0,00% |
| Interpretation of overlap | **Slight overlap** | |
| Structural Zeros | X | 0 |
| Corrected covered area  (adjusting by structural zeros) | (N-r)/(rc-r-X) | 0,00% |

MVPA. Obese or overweight people

| Overall results | | |
| --- | --- | --- |
|  |  |  |
| Number of columns (number of reviews) | c | 3 |
| Number of rows (number of index publications) | r | 20 |
| Number of included primary studies (including double counting) | N | 21 |
| Covered area | N/(rc) | 35,00% |
| Corrected covered area | (N-r)/(rc-r) | 2,50% |
| Interpretation of overlap | Slight overlap | |
| Structural Zeros | X | 0 |
| Corrected covered area  (adjusting by structural zeros) | (N-r)/(rc-r-X) | 2,50% |

## Figure S3.Physical activity as composite measurements

Composite measurements. Mixed population

| Overall results | | |
| --- | --- | --- |
|  |  |  |
| Number of columns (number of reviews) | c | 7 |
| Number of rows (number of index publications) | r | 157 |
| Number of included primary studies (including double counting) | N | 200 |
| Covered area | N/(rc) | 18,20% |
| Corrected covered area | (N-r)/(rc-r) | 4,56% |
| Interpretation of overlap | Slight overlap | |
| Structural Zeros | X | 0 |
| Corrected covered area  (adjusting by structural zeros) | (N-r)/(rc-r-X) | 4,56% |

Composite measurements. People with pathologies

| Overall results | | |
| --- | --- | --- |
|  |  |  |
| Number of columns (number of reviews) | c | 6 |
| Number of rows (number of index publications) | r | 72 |
| Number of included primary studies (including double counting) | N | 83 |
| Covered area | N/(rc) | 19,21% |
| Corrected covered area | (N-r)/(rc-r) | 3,06% |
| Interpretation of overlap | Slight overlap | |
| Structural Zeros | X | 0 |
| Corrected covered area  (adjusting by structural zeros) | (N-r)/(rc-r-X) | 3,06% |

## Figure S4. Sedentary Behaviour

Sedentary Behaviour. Mixed population

| Overall results | | |
| --- | --- | --- |
|  |  |  |
| Number of columns (number of reviews) | c | 5 |
| Number of rows (number of index publications) | r | 64 |
| Number of included primary studies (including double counting) | N | 75 |
| Covered area | N/(rc) | 23,44% |
| Corrected covered area | (N-r)/(rc-r) | 4,30% |
| Interpretation of overlap | Slight overlap | |
| Structural Zeros | X | 0 |
| Corrected covered area  (adjusting by structural zeros) | (N-r)/(rc-r-X) | 4,30% |

# Supplementary File 10. Meta-analyses effects sizes with certainty of evidence and AMSTAR 2 ratings

## Table S1. Physical activity as Steps per day

| WD VS ANY control | Imprecision cut off | Risk of bias (trial quality) | Inconsistency | Risk of bias (review quality*) | Certainty of evidence | Measure effect | Direction | Overall quality AMSTAR 2 |
| --- | --- | --- | --- | --- | --- | --- | --- | --- |
| Mixed population | | | | | | | | |
| Bravata, 2007 | >200 | NR | >75% | very serious limitation | NA | MD 2491 (1098 to 3885) | favour intervention | CL |
| Brickwood, 2019 | >200 | >75% low ROB | <75% | serious limitation | MODERATE | SMD 0.23 (0.15 to 0.32) | favour intervention | CL |
| Chaudhry, 2020 | >200 | <75% low ROB | >75% | serious limitation | LOW | MD 1854 (1217 to 2492) | favour intervention | L |
| Gal, 2018 | >200 | >75% low ROB | >75% | serious limitation | MODERATE | SMD 0.51 (0.12 to 0.91) | favour intervention | L |
| Lynch, 2020 | >200 | >75% low ROB | <75% | very serious limitation | MODERATE | SMD 0.25 (0.17 to 0.32) | favour intervention | CL |
| Tang, 2020 | >200 | >75% low ROB | <75% | very serious limitation | MODERATE | SMD 0.33 (0.161 to 0.504) | favour intervention | CL |
| People with pathologies | | | | | | | | |
| Armstrong, 2019 | >200 | >75% low ROB | >75% | serious limitation | MODERATE | SMD 0.53 (0.29 to 0.77) | favour intervention | CL |
| Ashur, 2021 | >200 | <75% low ROB | <75% | very serious limitation | LOW | MD 2587 (916 to 4257) | favour intervention | CL |
| Franssen, 2020 | >200 | >75% low ROB | >75% | serious limitation | MODERATE | MD 2123 (1605 to 2641) | favour intervention | CL |
| Hannan, 2019 | >200 | <75% low ROB | >75% | very serious limitation | LOW | SMD 0.45 (-0.17 to 1.07) | no difference | CL |
| Kamei, 2022 | >200 | >75% low ROB | <75% | very serious limitation | MODERATE | MD 333.48 (–415.83 to 1082.79) | no difference | CL |
| Kirk, 2019 | >200 | <75% low ROB | >75% | very serious limitation | LOW | MD 2592 (1688.62 to 3496.04) | favour intervention | CL |
| Lynch, 2018 | <200 | >75% low ROB | <75% | No serious limitation | MODERATE | MD 1400 (-40 to 2840) | no difference | M |
| Pudkasam, 2021 | >200 | >75% low ROB | <75% | serious limitation | MODERATE | SMD 0.16 (0.02 to 0.29) | favour intervention | L |
| Qiu, 2014 | >200 | <75% low ROB | >75% | very serious limitation | LOW | MD 1822 (751 to 2894) | favour intervention | CL |
| Older adults |  |  |  |  |  |  |  |  |
| Liu, 2020 | >200 | <75% low ROB | >75% | very serious limitation | LOW | SMD 1.27 (0.51 to 2.04) | favour intervention | CL |
| S Oliveira, 2020 | >200 | >75% low ROB | >75% | serious limitation | MODERATE | MD 1558 (1099 to 2018) | favour intervention | L |
| Yerrakalva, 2019 | >200 | >75% low ROB | >75% | very serious limitation | LOW | MD 506 (−80 to 1092) | no difference | L |
| Obese or overweight people |  |  |  |  |  |  |  |  |
| Wong, 2022 | <200 | >75% low ROB | <75% | serious limitation | MODERATE | MD 1243.51 (111.51 to 2375.51) | favour intervention | L |
| De Vries, 2016 | >200 | >75% low ROB | <75% | serious limitation | MODERATE | SMD 0.90 (0.61 to 1.19) | favour intervention | CL |
| Dehghan Ghahfarokhi, 2022 | >200 | >75% low ROB | >75% | very serious limitation | LOW | SMD 0.53 (0.24 to 0.82) | favour intervention | CL |

**Legend:** MD, mean difference; NA, not available; ROB, risk of bias; SMD, standardize mean difference; WD, wearable devices

**Note:**

- ‘High confidence’ (0-1 non-critical weakness), the SR provides an accurate and comprehensive summary of the results of the available studies that address the question of interest;
- ‘Moderate confidence’ (> 1 non-critical weakness), the SR has more than one weakness but no critical flaws. It may provide an accurate summary of the results of the available studies that were included in the review;
- ‘Low confidence’ (1 critical flaw with or without non-critical weaknesses), the SR has a critical flaw and may not provide an accurate and comprehensive summary of the available studies that address the question of interest; and
- ‘Critically low confidence’ (> 1 critical flaw with or without non-critical weaknesses): the SR has more than one critical flaw and should not be relied on to provide an accurate and comprehensive summary of the available studies.

## Table S2. Physical activity as moderate to Vigorous Physical Activity (MVPA)

| WD VS ANY control | Imprecision cut off | Risk of bias (trial quality) | Inconsistency | Risk of bias (review quality*) | Certainty of evidence | Measure effect | Direction | Overall quality AMSTAR 2 |
| --- | --- | --- | --- | --- | --- | --- | --- | --- |
| Mixed population | | | | | | | | |
| Brickwood, 2019 | >200 | >75% low ROB | <75% | serious limitation | MODERATE | SMD 0.28 (0.14 to 0.41) | favour intervention | CL |
| Gal, 2018 | >200 | >75% low ROB | >75% | serious limitation | MODERATE | SMD 0.43 (0.03 to 0.82) | favour intervention | L |
| Larsen, 2019 | >200 | <75% low ROB | <75% | No serious limitation | MODERATE | MD 0.34 (0.15 to 0.52) | favour intervention | L |
| Larsen, 2022 | >200 | <75% low ROB | <75% | serious limitation | MODERATE | SMD 0.23 (0.16 to 0.30) | favour intervention | L |
| Lynch, 2020 | >200 | >75% low ROB | NA | very serious limitation | NA | SMD -0.01 (-0.15 to 0.13) | no difference | CL |
| People with pathologies | | | | | | | | |
| Kirk, 2019 | >200 | <75% low ROB | >75% | very serious limitation | LOW | MD 36.31 (18.33 to 54.29) | favour intervention | CL |
| Lynch, 2018 | <200 | >75% low ROB | NA | No serious limitation | NA | MD 4.4 (0.28 to 8.52) | favour intervention | M |
| Pudkasam, 2021 | >200 | >75% low ROB | >75% | serious limitatation | MODERATE | SMD 0.55 (0.30 to 0.79) | favour intervention | L |
|  |  |  |  |  |  |  |  |  |
| Obese or overweight people |  |  |  |  |  |  |  |  |
| Wong, 2022 | >200 | >75% low ROB | <75% | serious limitation | MODERATE | MD 7.22 (2.84 to 11.61) | favour intervention | L |
| de Vries, 2016 | >200 | >75% low ROB | <75% | serious limitation | MODERATE | SMD 0.50 (0.11 to 0.88) | favour intervention | CL |
| Dehghan Ghahfarokhi, 2022 | >200 | >75% low ROB | >75% | very serious limitation | LOW | SMD 0.47 (0.06 to 0.88) | favour intervention | CL |
| Older adults | | | | | | | |  |
| Liu, 2020 | <200 | <75% low ROB | <75% | very serious limitation | LOW | SMD 1.23 (0.75 to 1.70) | favour intervention | CL |

**Legend:** MD, mean difference; NA, not available; ROB, risk of bias; SMD, standardize mean difference; WD, wearable device

##

## Table S3. Physical activity as composite measurements

| WD VS ANY control | Imprecision cut off | Risk of bias (trial quality) | Inconsistency | Risk of bias (review quality*) | Certainty of evidence | Measure effect | Direction | Overall quality AMSTAR 2 |
| --- | --- | --- | --- | --- | --- | --- | --- | --- |
| Mixed population | | | | | | | | |
| Braakhuis, 2019 | >200 | <75% low ROB | <75% | very serious limitation | LOW | SMD 0.34 (0.23 to 0.44) | favour intervention | CL |
| Gierisch, 2015 | >200 | <75% low ROB | <75% | very serious limitation | LOW | SMD 0.26 (0.04 to 0.49) | favour intervention | CL |
| Goode, 2017 | >200 | <75% low ROB | <75% | very serious limitation | LOW | SMD 0.26 (0.04 to 0.49) | favour intervention | CL |
| Laranjo, 2021 | >200 | <75% low ROB | <75% | very serious limitation | LOW | SMD 0.350 (0.236 to 0.465) | favour intervention | M |
| Larsen, 2019 | >200 | <75% low ROB | >75% | no serious limitation | MODERATE | SMD 0.53 (0.34 to 0.73) | favour intervention | L |
| Larsen, 2022 | >200 | <75% low ROB | >75% | serious limitation | LOW | SMD 0.42 (0.28 to 0.55) | favour intervention | L |
| Tang, 2020 | >200 | >75% low ROB | >75% | very serious limitation | LOW | SMD 0.449 (0.102 to 0.796) | favour intervention | CL |
| People with pathologies | | | | | | | | |
| Baskerville, 2017 | >200 | <75% low ROB | >75% | very serious limitation | LOW | SMD 0.57 (0.24 0.91) | favour intervention | CL |
| de Leeuwerk, 2022 | >200 | >75% low ROB | <75% | very serious limitation | MODERATE | SMD 0.34 (0.12 to 0.56) | favour intervention | CL |
| Hodkinson, 2021 | >200 | <75% low ROB | >75% | very serious limitation | LOW | SMD 0.72 (0.46 to 0.97) | favour intervention | CL |
| Qiu, 2018 | >200 | <75% low ROB | >75% | very serious limitation | LOW | SMD 0.57 (0.31 to 0.84) | favour intervention | CL |
| Rintala, 2018 | >200 | >75% low ROB | <75% | very serious limitation | MODERATE | SMD 0.59 (0.38 to 0.79) | favour intervention | CL |
| Vaes, 2013 | >200 | >75% low ROB | <75% | very serious limitation | MODERATE | SMD 0.81 (0.46 to 1.17) | favour intervention | CL |
| Older adults |  |  |  |  |  |  |  |  |
| Cooper, 2018 | >200 | >75% low ROB | <75% | very serious limitation | MODERATE | SMD 0.22 (-0.08 to 0.51) | no difference | CL |

Legend; MD, mean difference; NA, not available; ROB, risk of bias; SMD, standardize mean difference; WD, wearable devices

## Table S4. Sedentary behaviour

| WD VS ANY control | Imprecision cut off | Risk of bias (trial quality) | Inconsistency | Risk of bias (review quality*) | Certainty of evidence | Measure effect | Direction | Overall quality AMSTAR 2 |
| --- | --- | --- | --- | --- | --- | --- | --- | --- |
| Mixed population | | | | | | | | |
| Brickwood, 2019 | >200 | >75% low ROB | <75% | serious limitation | MODERATE | SMD −0.20 (−0.43 to 0.03) | no difference | CL |
| Larsen, 2019 | <200 | <75% low ROB | <75% | no serious limitation | MODERATE | SMD -0.40 (-1.07 to 0.27) | no difference | L |
| Larsen, 2022 | >200 | <75% low ROB | <75% | serious limitation | MODERATE | SMD -0.12 (-0.25 to 0.01) | no difference | L |
| Qiu, 2015 | >200 | <75% low ROB | <75% | serious limitation | MODERATE | SMD -0.20 (-0.33 to -0.07) | favour intervention | CL |
| Stephenson, 2017 | >200 | >75% low ROB | >75% | very serious limitation | MODERATE | MD -41.28 (-60.99 to -21.58) | favour intervention | CL |
| Older adults | | | | | | | | |
| Yerrakalva, 2019 | <200 | >75% low ROB | <75% | very serious limitation | LOW | SMD −0.49 (−1.02 to 0.03) | no difference | L |

**Legend:** MD, mean difference; NA, not available; ROB, risk of bias; SMD, standardize mean difference; WD, wearable devices

# Supplementary File 11. Bubble plots linking Certainty of Evidence with direction of effect

## Figure S1. Map of evidence


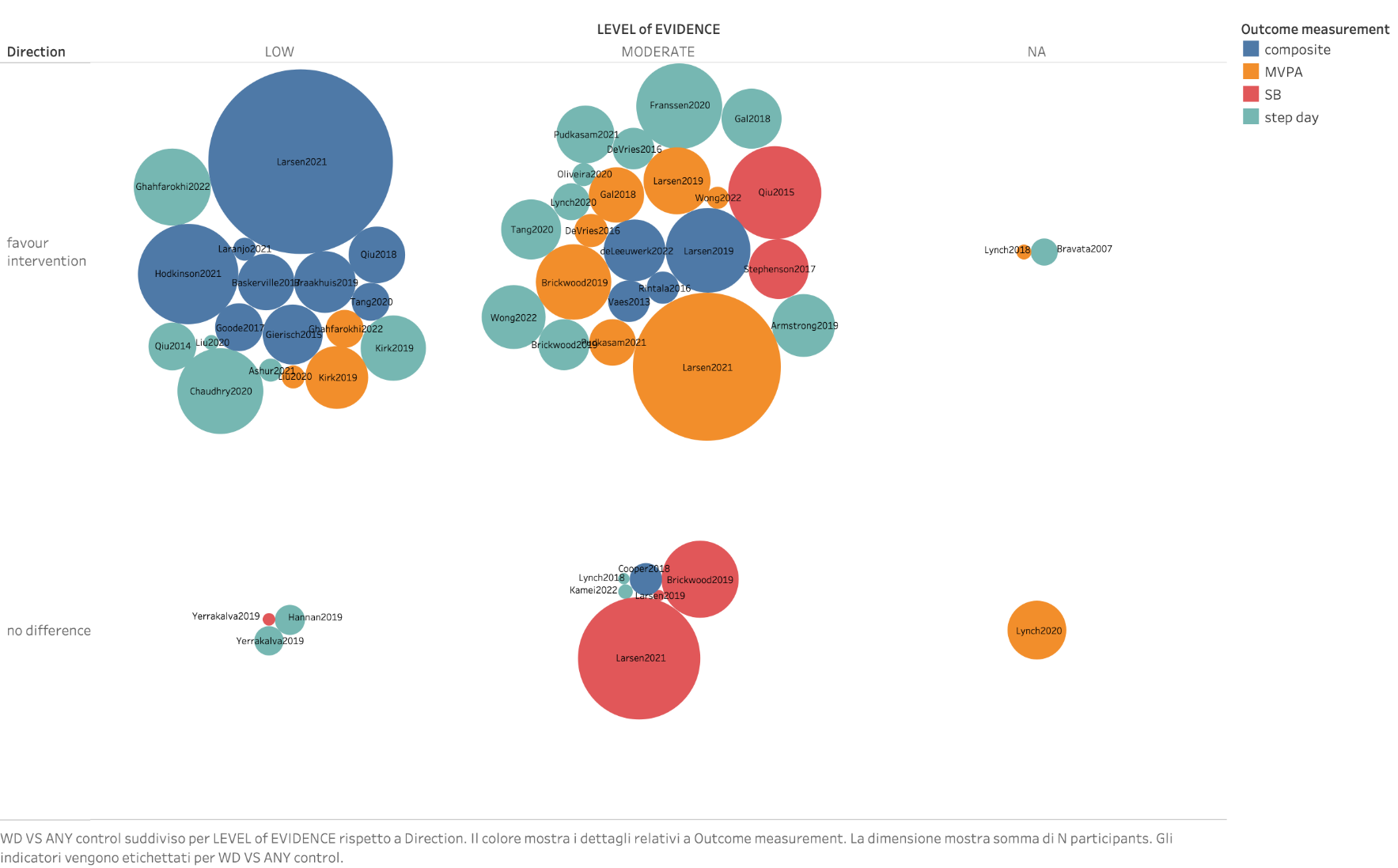


**Legend.** This graphic provides information in three dimensions: (1) in the y-axis, there is the rating of authors conclusions as “beneficial for intervention”, “no effect” and “beneficial for control” (they were further described in the data collection section); (2) in the x-axis, the GRADE assessment is shown; and (3) we displayed the bubble size proportionally to the number of participants included in each SR.

MVPA, moderate to vigorous physical activity; SB, sedentary behaviour. Step per day is measured in number of steps, MVPA and SB are measured in minutes, composite measurements is measured in standardized mean difference (SMD) as standardized measure of physical activity (e.g., metabolic equivalent for task (MET), min/week, intensity, time spent walking)

## Figure S2. Physical activity as steps per day


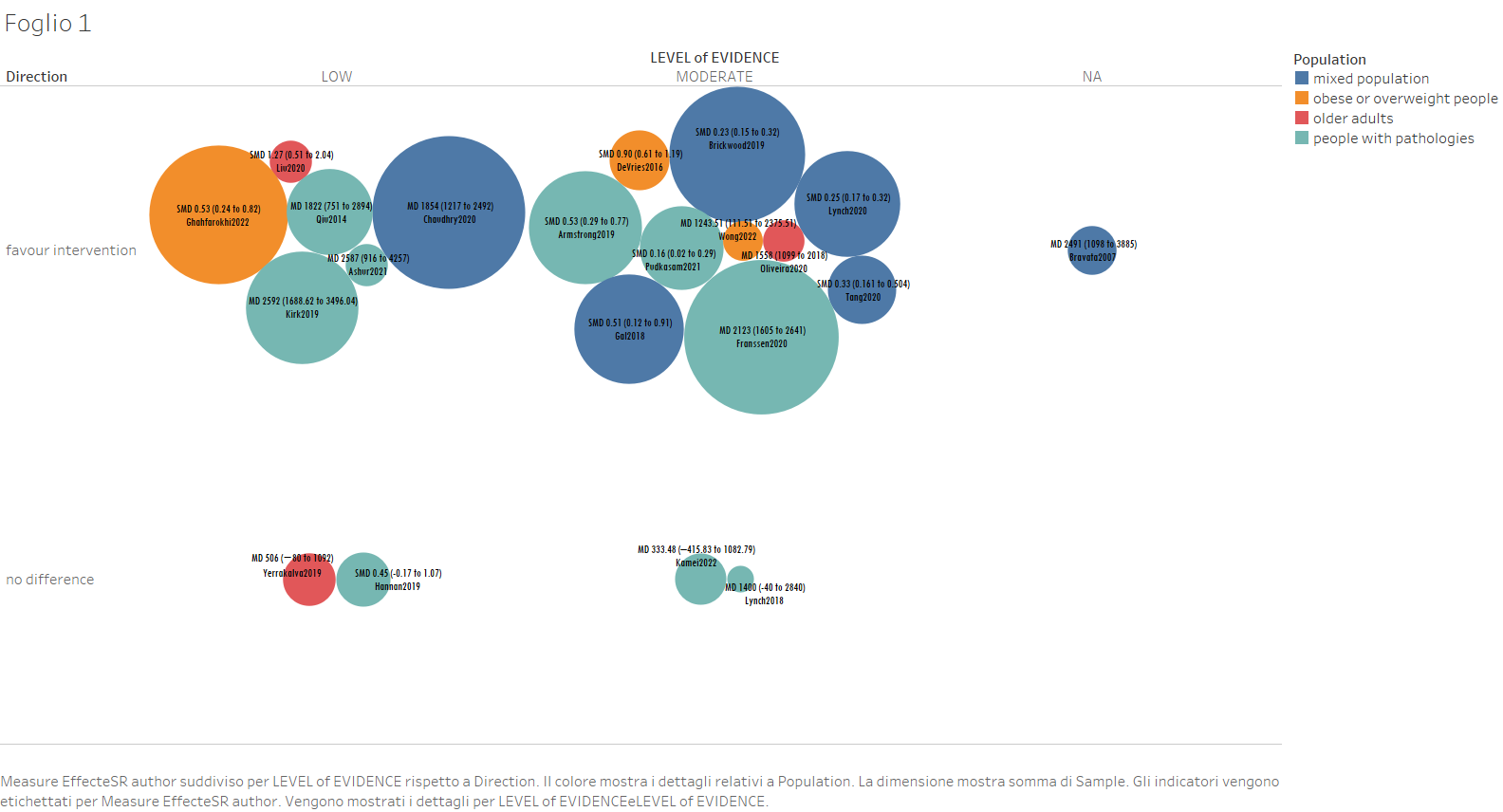


## Figure S3. Physical activity as Moderate to Vigorous Physical Activity (MPVA)


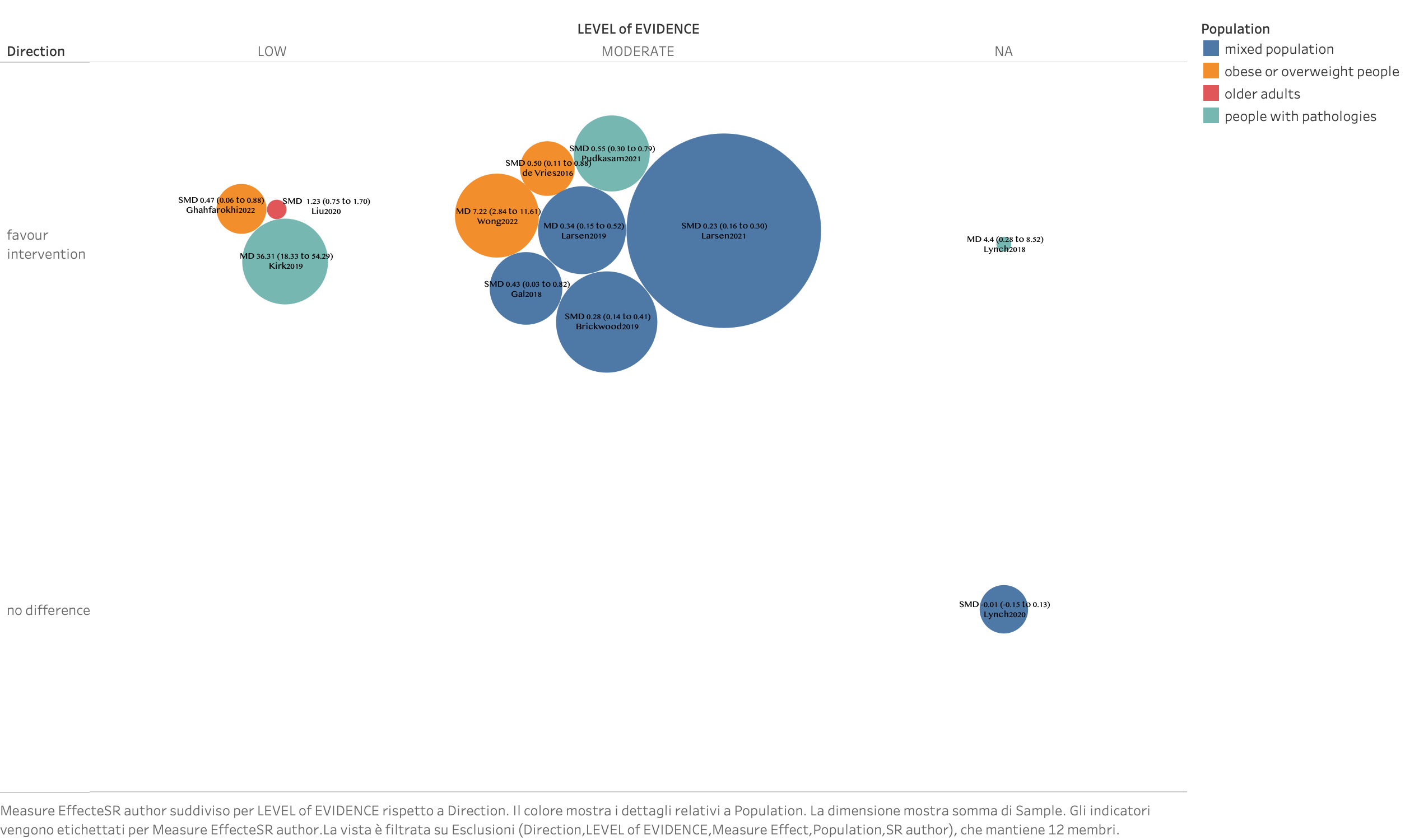


## Figure S4. Physical activity as composite outcome


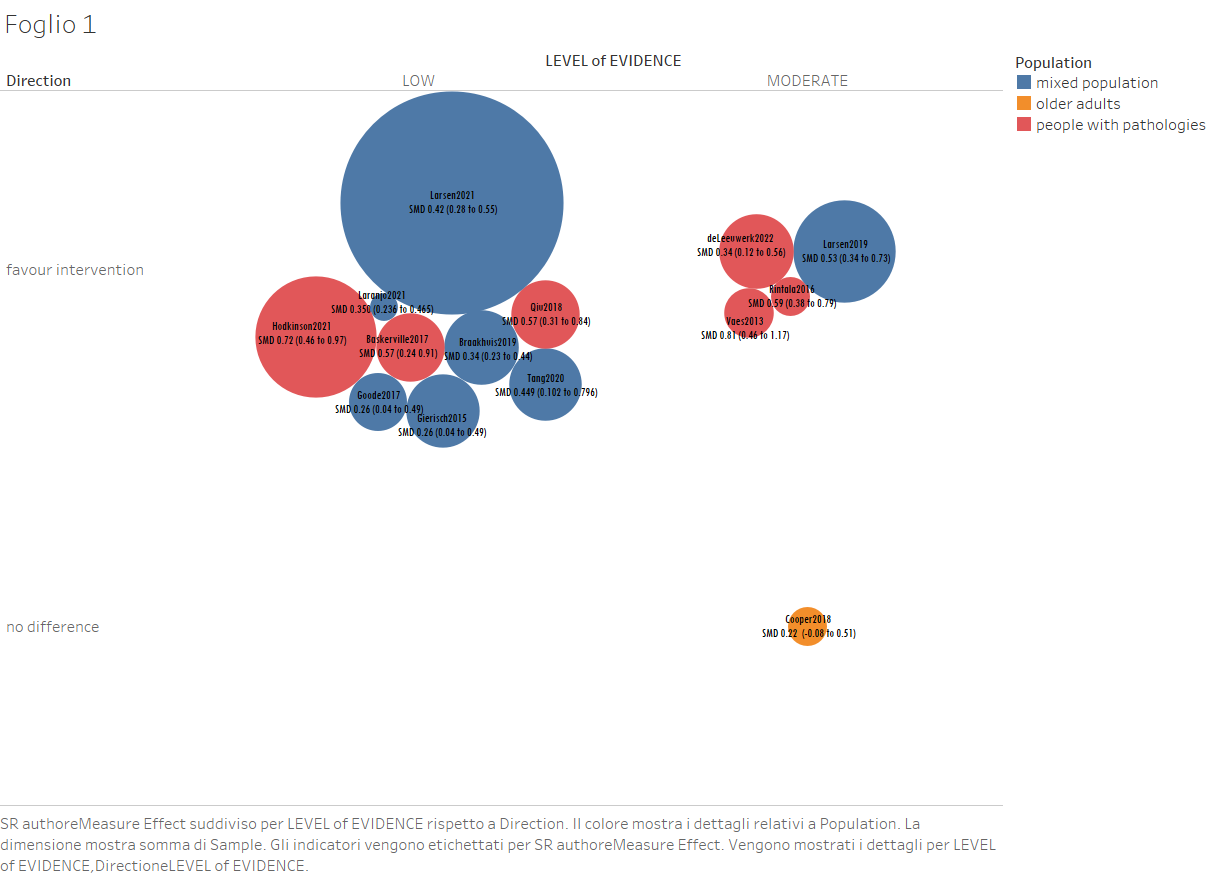


## Figure S5. Sedentary behavior


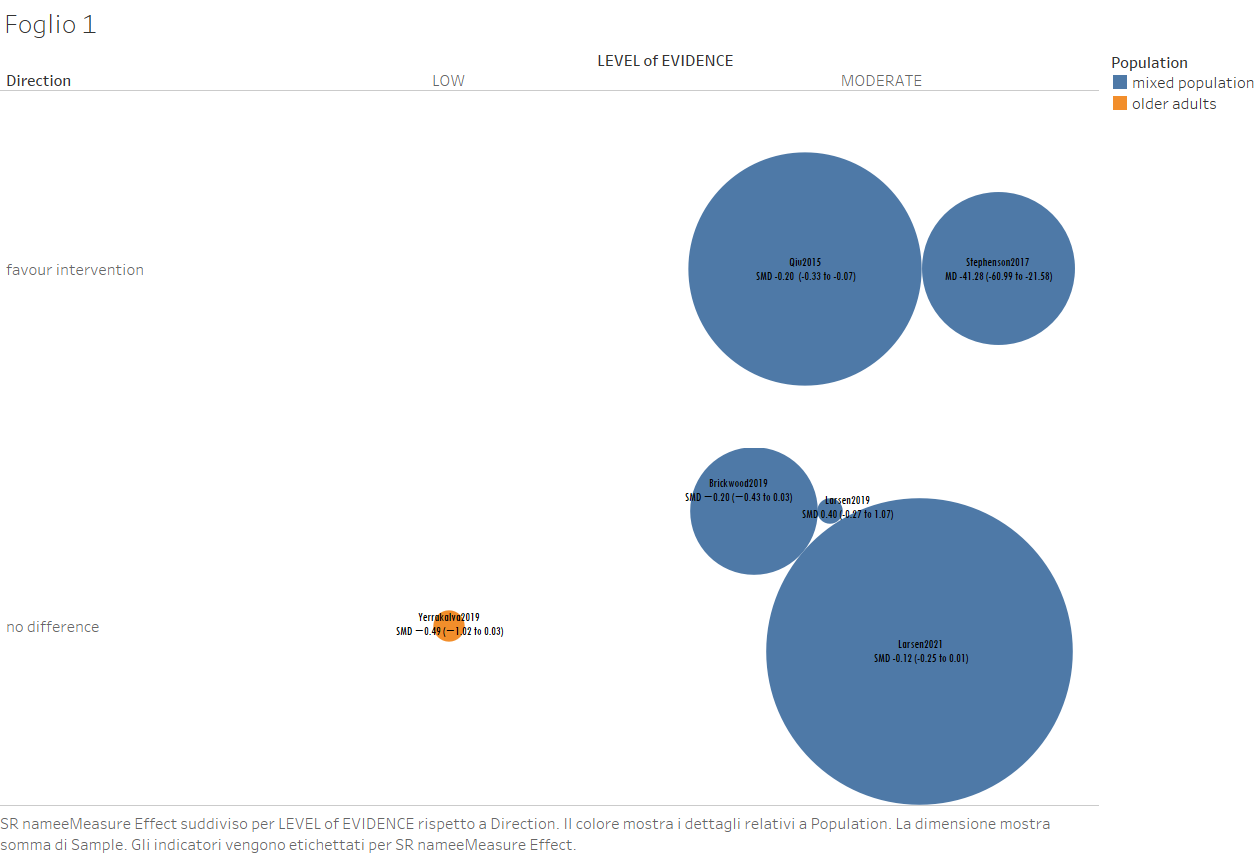


# Supplementary File 12. Clinical relevance

## Figure S1. Plotting Measure Effects – Sedentary behaviour


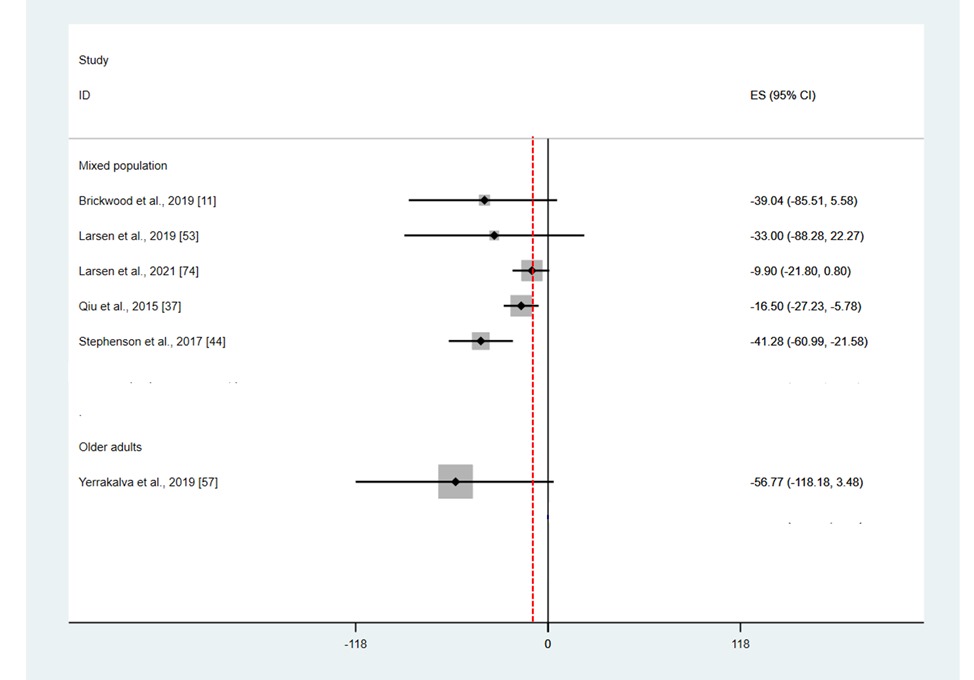


**Legend:** red line refers to the clinical relevance (Larsen 2021 [31]).

## Figure S2. Plotting Measure Effects – Composite measurements

**

Larsen 2021 back translated SMD 0.42 (0.28, 055) into 1235 (95% CI 823 to 1617) daily steps

## Table S1. Clinical relevance assessment

|  | Steps per day | | MVPA | | SB | |
| --- | --- | --- | --- | --- | --- | --- |
|  | N of Systematic review | Percentage of Systematic review | N of Systematic review | Percentage of Systematic review | N of Systematic review | Percentage of Systematic review |
| Definitive | 3 | 14.29 | 3 | 25.0 | 1 | 16.67 |
| Probable yes | 8 | 38.10 | 5 | 41.7 | 1 | 16.67 |
| Probable not | 1 | 4.76 |  |  | 4 | 66.67 |
| Possible yes | 2 | 9.52 | 3 | 25 |  |  |
| Possible no | 2 | 9.52 |  |  |  |  |
| Definitive not | 4 | 19.05 | 1 | 8.3 |  |  |
| Definitive not no | 1 | 4.76 |  |  |  |  |

**Legend:** n, number; MPVA, Moderate to Vigorous Physical Activity; SB, Sedentary behavior.

# References

1. Aromataris E, Fernandez R, Godfrey C, Holly C, Khalil H, Bhatarasakoon P. Chapter 10: Umbrella Reviews. 2020.

2. Pollock M, Fernandes R, Becker L, Pieper D, Hartling L. Part 1, Chapter V: Overviews of Reviews. . In: Julian Higgins JT, editor. Cochrane Handbook for Systematic Reviews of Interventions Handbook; 2022.

3. Page MJ, McKenzie JE, Bossuyt PM, Boutron I, Hoffmann TC, Mulrow CD, et al. The PRISMA 2020 statement: an updated guideline for reporting systematic reviews. Bmj. 2021 Mar 29;372:n71.

4. Pollock M, Fernandes RM, Pieper D, Tricco AC, Gates M, Gates A, et al. Preferred Reporting Items for Overviews of Reviews (PRIOR): a protocol for development of a reporting guideline for overviews of reviews of healthcare interventions. Syst Rev. 2019 Dec 23;8(1):335.

5. Gates M, Gates A, Pieper D, Fernandes RM, Tricco AC, Moher D, et al. Reporting guideline for overviews of reviews of healthcare interventions: development of the PRIOR statement. BMJ. 2022 Aug 9;378:e070849.

6. Shea BJ, Reeves BC, Wells G, Thuku M, Hamel C, Moran J, et al. AMSTAR 2: a critical appraisal tool for systematic reviews that include randomised or non-randomised studies of healthcare interventions, or both. Bmj. 2017 Sep 21;358:j4008.

7. Pollock A, Farmer SE, Brady MC, Langhorne P, Mead GE, Mehrholz J, et al. An algorithm was developed to assign GRADE levels of evidence to comparisons within systematic reviews. J Clin Epidemiol. 2016 Feb;70:106-10.

8. Schünemann HJ OA, Vist GE, Higgins JP, Deeks JJ, Glasziou P, et al. . Chapter 12: Interpreting results and drawing conclusions. In: Higgins JP, Green S, editor(s). Cochrane Handbook for Systematic Reviews of Interventions Version 5.1.0 (updated March 2011). The Cochrane Collaboration, 2011. Available from handbook.cochrane.org.

1. Aromataris E, Fernandez R, Godfrey C, Holly C, Khalil H, Bhatarasakoon P. Chapter 10: Umbrella Reviews. In:2020.

2. Pollock M, Fernandes R, Becker L, Pieper D, Hartling L. Part 1, Chapter V: Overviews of Reviews. . In: Julian Higgins JT, ed. *Cochrane Handbook for Systematic Reviews of Interventions Handbook.*2022.

3. Page MJ, McKenzie JE, Bossuyt PM, et al. The PRISMA 2020 statement: an updated guideline for reporting systematic reviews. *Bmj.* 2021;372:n71.

4. Pollock M, Fernandes RM, Pieper D, et al. Preferred Reporting Items for Overviews of Reviews (PRIOR): a protocol for development of a reporting guideline for overviews of reviews of healthcare interventions. *Syst Rev.* 2019;8(1):335.

5. Gates M, Gates A, Pieper D, et al. Reporting guideline for overviews of reviews of healthcare interventions: development of the PRIOR statement. *BMJ.* 2022;378:e070849.

6. Shea BJ, Reeves BC, Wells G, et al. AMSTAR 2: a critical appraisal tool for systematic reviews that include randomised or non-randomised studies of healthcare interventions, or both. *Bmj.* 2017;358:j4008.

7. Pollock A, Farmer SE, Brady MC, et al. An algorithm was developed to assign GRADE levels of evidence to comparisons within systematic reviews. *J Clin Epidemiol.* 2016;70:106-110.

8. Schünemann HJ OA, Vist GE, Higgins JP, Deeks JJ, Glasziou P, et al. . Chapter 12: Interpreting results and drawing conclusions. In: Higgins JP, Green S, editor(s). Cochrane Handbook for Systematic Reviews of Interventions Version 5.1.0 (updated March 2011). The Cochrane Collaboration, 2011. Available from handbook.cochrane.org.

1. Aromataris E, Fernandez R, Godfrey C, Holly C, Khalil H, Bhatarasakoon P. Chapter 10: Umbrella Reviews. In:2020.

2. Pollock M, Fernandes R, Becker L, Pieper D, Hartling L. Part 1, Chapter V: Overviews of Reviews. . In: Julian Higgins JT, ed. *Cochrane Handbook for Systematic Reviews of Interventions Handbook.*2022.

3. Page MJ, McKenzie JE, Bossuyt PM, et al. The PRISMA 2020 statement: an updated guideline for reporting systematic reviews. *Bmj.* 2021;372:n71.

4. Pollock M, Fernandes RM, Pieper D, et al. Preferred Reporting Items for Overviews of Reviews (PRIOR): a protocol for development of a reporting guideline for overviews of reviews of healthcare interventions. *Syst Rev.* 2019;8(1):335.

5. Gates M, Gates A, Pieper D, et al. Reporting guideline for overviews of reviews of healthcare interventions: development of the PRIOR statement. *BMJ.* 2022;378:e070849.

6. Shea BJ, Reeves BC, Wells G, et al. AMSTAR 2: a critical appraisal tool for systematic reviews that include randomised or non-randomised studies of healthcare interventions, or both. *Bmj.* 2017;358:j4008.

7. Pollock A, Farmer SE, Brady MC, et al. An algorithm was developed to assign GRADE levels of evidence to comparisons within systematic reviews. *J Clin Epidemiol.* 2016;70:106-110.

8. Schünemann HJ OA, Vist GE, Higgins JP, Deeks JJ, Glasziou P, et al. . Chapter 12: Interpreting results and drawing conclusions. In: Higgins JP, Green S, editor(s). Cochrane Handbook for Systematic Reviews of Interventions Version 5.1.0 (updated March 2011). The Cochrane Collaboration, 2011. Available from handbook.cochrane.org.

1. Aromataris E, Fernandez R, Godfrey C, Holly C, Khalil H, Bhatarasakoon P. Chapter 10: Umbrella Reviews. In:2020.

2. Pollock M, Fernandes R, Becker L, Pieper D, Hartling L. Part 1, Chapter V: Overviews of Reviews. . In: Julian Higgins JT, ed. *Cochrane Handbook for Systematic Reviews of Interventions Handbook.*2022.

3. Page MJ, McKenzie JE, Bossuyt PM, et al. The PRISMA 2020 statement: an updated guideline for reporting systematic reviews. *Bmj.* 2021;372:n71.

4. Pollock M, Fernandes RM, Pieper D, et al. Preferred Reporting Items for Overviews of Reviews (PRIOR): a protocol for development of a reporting guideline for overviews of reviews of healthcare interventions. *Syst Rev.* 2019;8(1):335.

5. Gates M, Gates A, Pieper D, et al. Reporting guideline for overviews of reviews of healthcare interventions: development of the PRIOR statement. *BMJ.* 2022;378:e070849.

6. Higgins J, Thomas J, Chandler JC, M, Li T, Page M, Welch V. Cochrane Handbook for Systematic Reviews of Interventions version 6.3 (updated February 2022)2022, Available from www.training.cochrane.org/handbook.

7. Ouzzani M, Hammady H, Fedorowicz Z, Elmagarmid A. Rayyan - a web and mobile app for systematic reviews. *Systematic Reviews.* 2016;5(1):210.

8. Edwards P, Clarke M, DiGuiseppi C, Pratap S, Roberts I, Wentz R. Identification of randomized controlled trials in systematic reviews: accuracy and reliability of screening records. *Stat Med.* 2002;21(11):1635-1640.

9. Shea BJ, Reeves BC, Wells G, et al. AMSTAR 2: a critical appraisal tool for systematic reviews that include randomised or non-randomised studies of healthcare interventions, or both. *Bmj.* 2017;358:j4008.

10. Pollock A, Farmer SE, Brady MC, et al. An algorithm was developed to assign GRADE levels of evidence to comparisons within systematic reviews. *J Clin Epidemiol.* 2016;70:106-110.

11. Schünemann HJ OA, Vist GE, Higgins JP, Deeks JJ, Glasziou P, et al. . Chapter 12: Interpreting results and drawing conclusions. In: Higgins JP, Green S, editor(s). Cochrane Handbook for Systematic Reviews of Interventions Version 5.1.0 (updated March 2011). The Cochrane Collaboration, 2011. Available from handbook.cochrane.org.

1. Higgins J, Thomas J, Chandler JC, M, Li T, Page M, Welch V. Cochrane Handbook for Systematic Reviews of Interventions version 6.3 (updated February 2022)2022, Available from www.training.cochrane.org/handbook.

2. Ouzzani M, Hammady H, Fedorowicz Z, Elmagarmid A. Rayyan - a web and mobile app for systematic reviews. *Systematic Reviews.* 2016;5(1):210.

3. Edwards P, Clarke M, DiGuiseppi C, Pratap S, Roberts I, Wentz R. Identification of randomized controlled trials in systematic reviews: accuracy and reliability of screening records. *Stat Med.* 2002;21(11):1635-1640.

4. Shea BJ, Reeves BC, Wells G, et al. AMSTAR 2: a critical appraisal tool for systematic reviews that include randomised or non-randomised studies of healthcare interventions, or both. *Bmj.* 2017;358:j4008.

5. Pollock A, Farmer SE, Brady MC, et al. An algorithm was developed to assign GRADE levels of evidence to comparisons within systematic reviews. *J Clin Epidemiol.* 2016;70:106-110.

6. Schünemann HJ OA, Vist GE, Higgins JP, Deeks JJ, Glasziou P, et al. . Chapter 12: Interpreting results and drawing conclusions. In: Higgins JP, Green S, editor(s). Cochrane Handbook for Systematic Reviews of Interventions Version 5.1.0 (updated March 2011). The Cochrane Collaboration, 2011. Available from handbook.cochrane.org.

1. Higgins J, Thomas J, Chandler JC, M, Li T, Page M, Welch V. Cochrane Handbook for Systematic Reviews of Interventions version 6.3 (updated February 2022)2022, Available from www.training.cochrane.org/handbook.

2. Shea BJ, Reeves BC, Wells G, et al. AMSTAR 2: a critical appraisal tool for systematic reviews that include randomised or non-randomised studies of healthcare interventions, or both. *Bmj.* 2017;358:j4008.

3. Pollock A, Farmer SE, Brady MC, et al. An algorithm was developed to assign GRADE levels of evidence to comparisons within systematic reviews. *J Clin Epidemiol.* 2016;70:106-110.

4. Schünemann HJ OA, Vist GE, Higgins JP, Deeks JJ, Glasziou P, et al. . Chapter 12: Interpreting results and drawing conclusions. In: Higgins JP, Green S, editor(s). Cochrane Handbook for Systematic Reviews of Interventions Version 5.1.0 (updated March 2011). The Cochrane Collaboration, 2011. Available from handbook.cochrane.org.

1. Shea BJ, Reeves BC, Wells G, et al. AMSTAR 2: a critical appraisal tool for systematic reviews that include randomised or non-randomised studies of healthcare interventions, or both. *Bmj.* 2017;358:j4008.

2. Pollock A, Farmer SE, Brady MC, et al. An algorithm was developed to assign GRADE levels of evidence to comparisons within systematic reviews. *J Clin Epidemiol.* 2016;70:106-110.

3. Schünemann HJ OA, Vist GE, Higgins JP, Deeks JJ, Glasziou P, et al. . Chapter 12: Interpreting results and drawing conclusions. In: Higgins JP, Green S, editor(s). Cochrane Handbook for Systematic Reviews of Interventions Version 5.1.0 (updated March 2011). The Cochrane Collaboration, 2011. Available from handbook.cochrane.org.

1. Shea BJ, Reeves BC, Wells G, et al. AMSTAR 2: a critical appraisal tool for systematic reviews that include randomised or non-randomised studies of healthcare interventions, or both. *Bmj.* 2017;358:j4008.

2. Pollock A, Farmer SE, Brady MC, et al. An algorithm was developed to assign GRADE levels of evidence to comparisons within systematic reviews. *J Clin Epidemiol.* 2016;70:106-110.

1. Shea BJ, Reeves BC, Wells G, et al. AMSTAR 2: a critical appraisal tool for systematic reviews that include randomised or non-randomised studies of healthcare interventions, or both. *Bmj.* 2017;358:j4008.

1. Shea BJ, Reeves BC, Wells G, Thuku M, Hamel C, Moran J, et al. AMSTAR 2: a critical appraisal tool for systematic reviews that include randomised or non-randomised studies of healthcare interventions, or both. Bmj. 2017;358:j4008.
